# Biodiversity of *Eucalyptus* endophytic fungi across different climates in Iran

**DOI:** 10.1371/journal.pone.0345700

**Published:** 2026-07-23

**Authors:** Parmida Aleahmad, Leila Ebrahimi, Naser Safaie

**Affiliations:** 1 Department of Entomology and Plant Pathology, Faculty of Agricultural Technology, University of Tehran, Tehran, Iran; 2 Department of Plant Pathology, Faculty of Agriculture, Tarbiat Modares University, Tehran, Iran; Council of Scientific & Industrial Research Indian Institute of Integrative Medicine, INDIA

## Abstract

Endophytic fungi are crucial for plant health, but the relative importance of climate versus host factors in shaping their communities is not fully understood. This study investigates the hypothesis that macro-climate is one of the primary drivers of endophytic fungal diversity and composition in *Eucalyptus camaldulensis* across different regions in Iran. A total of 712 fungal isolates were isolated from leaves, branches, and fruits across five provinces (Alborz, Isfahan, Mazandaran, Qom, and Tehran) spanning arid to humid climates. Cultivation and morphological/molecular identification revealed a clear diversity gradient, with the highest species richness, Shannon diversity, and colonization frequency in the humid province of Mazandaran, and the lowest in the semi-arid and arid provinces of Alborz and Qom, respectively. Community composition shifted significantly along this climatic gradient: arid sites were dominated by stress-tolerant generalists (e.g., *Alternaria*), while the humid site supported a balanced community of specialists (e.g., *Neofusicoccum*). Across all climates, tissue type was a consistent secondary filter, with leaves harboring the most diverse communities, followed by branches and fruits. These results demonstrate a hierarchical assembly where climate filters the regional species pool, and host tissue type then fine-tunes the local community. This study provides the first comprehensive baseline of *E. camaldulensis* endophytes in Iran and highlights the vulnerability of these symbiotic systems to climate change, while also identifying them as a valuable resource for biotechnology.

## Introduction

Fungal endophytes are defined as a group of fungi that colonize living internal tissues of plants without causing any symptoms for the entire or at least a significant part of their life cycle [1]. Endophytic fungi are associated with different parts of plants such as seeds, roots, branches, leaves, fruits and flowers [2]. These microorganisms’ inhabit plants as a shelter and source of nutrition and in exchange they may benefit the host plants by enhancing growth and inducing resistance or tolerance to various biotic and abiotic stresses [3–5]. The term “endophytes” refer to a range of symbioses such as mutualism, commensalism, amensalism, dormant saprophytic, parasitism and even neutralism [6,7]. Host-endophytic associations vary from plant to plant due to different environmental factors influencing their adaptation and survival [8]. Different biotic factors such as plant age, host taxonomy identity, type of plant tissue, intra- and interspecific relationships, and abiotic factors including collection time (season), geographic factors, climate, availability of soil nutrients and moisture influence the endophytic mycobiota within the same plant species [9]. Endophytes exhibit greater diversity than plant pathogens within plant systems [10]. Almost every investigated plant demonstrates the presence of endophytes. Therefore, it has been hypothesized over one million endophytic species exist in 300,000 different plant species but only a small portion has been isolated and studied [11]. Every plant including trees, fruits, vegetables, cereal grains, and other crops harbors various fungal endophytes which primarily belong to Ascomycota, and also some taxa from Basidiomycota, Zygomycota, and Oomycota [12,13].

The genus *Eucalyptus*, endemic to the Australian region [14] and the second main industrial timber commodity [15], with over 747 species, is substantial due to rapid growth, high wood properties and wide ecological range [16]. *Eucalyptus* spp. are rich in flavonoids and phenolic, which present high antibacterial, cytotoxic, antifungal and allelopathic activities [17]. Fungal and bacterial endophytes associated with *E. globulus* [18–20]*, E. urophylla* x *E. grandis* [21], *E. grandis* x *E. urophylla* [22], *E. citriodora*, *E. grandis*, *E. urophylla*, *E. camaldulensis* Dehnh, *E. torelliana* and *E. pellita* [23], *E. clones* [24], *E. nitens* [25], *E. microcorys* [26], *E. exserta* [27] and other species have been investigated in different regions around the world.

In a study of endophytic fungi in *Eucalyptus* leaves and branches, Fisher et al. [25] found *Botryosphaeria dothidea* (Moug.) Ces. and De Not. and *Cytospora eucalypticola* Van der Westh. to be the most frequent species. Lupo et al. [19] isolated the species *Cytospora chrysosperma* (Pers.) Fr., *Fusicoccum eucalypti* Sousa da Câmara, *Alternaria alternata* (Fr.) Keissl., *Fairmaniella leprosa* (Fairm.) Petr. and Syd., *Aureobasidium pullulans* (de Bary and Löwenthal) G. Arnaud, and *Cladosporium cladosporioides* (Fresen.) G.A. de Vries as endophytes from the flowers, capsules, and seeds of *E. globulus* Labill. Lacerda et al. [26] studied the composition of fungal endophyte communities in *E. microcorys* and reported *Castanediella eucalypticola* Crous and M.J. Wingf. and *Neophaeomoniella eucalypti* Roon.-Lath. and Crous for the first time in Brazil. In an investigation of *E. globulus* twig endophytes, Fillat et al. [20] obtained 127 fungal isolates and identified several laccase-positive strains, including *Pringsheimia smilacis* E. Müll., *Lophiostoma corticola* (Fuckel) E.C.Y. Liew, Aptroot and K.D. Hyde, *Hormonema* sp., *Neofusicoccum luteum* (Pennycook and Samuels) Crous, Slippers and A.J.L. Phillips, *Phaeomoniella effusa* Damm and Crous, and *Ulocladium* sp.. In another study, Mao et al. [27] obtained 80 endophytic fungal isolates from *E. exserta* and identified 13 genera: *Penicillium* Link, *Chaetomium* Kunze, *Cladosporium* Link, *Phyllosticta* Pers., *Eutypella* (Nitschke) Sacc., *Purpureocillium* Luangsa-ard, Hywel-Jones, Houbraken and Samson, *Gongronella* Ribaldi, *Talaromyces* C.R. Benj., *Pestalotiopsis* Steyaert, *Fusarium* Link, *Lophiostoma* Ces. and De Not., *Scedosporium* Sacc. ex Castell. and Chalm., and *Pseudallescheria* Negr. and I. Fisch.

*Eucalyptus* is a globally vital genus, cultivated extensively for timber, pulp, and especially for its complex essential oils, which possess potent antimicrobial, anti-inflammatory, and antioxidant properties. Intriguingly, the endophytic fungi residing within plants are known to produce a similar array of bioactive compounds. Therefore, investigating the endophytic microbiome of *Eucalyptus* is crucial to discover novel fungal strains and metabolites for agricultural and pharmaceutical applications. While the endophytic mycobiota of *Eucalyptus* species have been studied in various parts of the world, no such investigation has been conducted in Iran, leaving a significant gap in our understanding of their associated fungi in this region. This study therefore investigated the hypothesis that macro-climatic conditions and host tissue type are the principal driver of endophytic fungal community structure in *E. camaldulensis*. To achieve this, we aimed to isolate and identify the culturable endophytic fungi from leaves, branches, and fruits of *E. camaldulensis* across different regions in Iran. By comparing diversity indices, community composition, and colonization frequencies across these different climates and tissues, we sought to determine the principal ecological factors governing the assembly of these hidden fungal communities and establish the first comprehensive baseline for their study in Iran.

## Materials and methods

### Plant material collection

Forty-five randomly selected *Eucalyptus camaldulensis* plant samples, including healthy and symptomless leaves, 1- or 2-year-old branches, and fruits, were collected from Alborz, Qom, Isfahan, Tehran, and Mazandaran provinces of Iran during the autumn of 2022. Climate information for each region is provided in S1 Table.

### Isolation of endophytic fungi

Endophytic fungi were isolated from plant samples using a modified Strobel and Daisy [28] procedure [29]. After surface sterilization with ethanol and sodium hypochlorite, disinfected plant tissues were cultured on water agar (WA) medium. As a control for the sterilization process, 100 µl of the final rinse water was plated on a WA medium. Emerging fungal hyphae were transferred to potato dextrose agar (PDA) for further studies.

### Morphological identification of endophytic fungi

Endophytic fungal isolates were characterized based on their macroscopic and microscopic morphological features, including colony shape and characteristics, colors, growth rate, asexual and sexual reproductive structures (if present). For morphological identification, fungal isolates were individually inoculated on PDA medium. Microscopic slides of the isolated endophytic fungi were prepared using lacto-phenol or lacto-phenol cotton blue solutions. Fungal isolates were identified to the genus level using valid keys, including those by Simmons [30], Ellis [31,32], Klich and Pitt [33], Klich [34], Sivanesan [35], Watanabe [36], Samuels [37,38], Crous [39], and Thambugala [40], Prokhorov and Linnik [41].

### Molecular identification of fungal endophytes

Endophytic fungal isolates were cultured on PDA medium in the dark at 25 °C for 7 days. DNA was extracted using the CTAB method [42], amplified for the Internal transcribed spacer region (ITS) using ITS1/ITS4 primers [43], and *Translation elongation factor 1-α* region (*tef-1α*) using EF1/EF2 primers [44], following PCR conditions from Ebrahimi and Fotouhifar [45]. Sequencing was performed at Noor Genetics Center, Iran. Obtained sequences were compared to related species using NCBI BLAST for taxonomic confirmation and submitted to BankIT (S2 Table).

### Diversity analysis of isolated endophytic fungi

Fungal diversity within *E. camaldulensis* tissues was assessed using two key metrics: species richness (the total number of fungal species present) and evenness (the distribution of individuals among species) [46]. The fungal community composition was quantified by recording the number of isolates (N) and species (S) obtained from each sample. Morphological characterization was performed through macroscopic examination to classify fungal morphotypes. In this context, “abundance” (N) refers to the total number of isolates, while “richness” (S) indicates the number of distinct species within the endophytic fungal community. These analyses were conducted on leaves, branches and fruit samples collected from *Eucalyptus* trees. To evaluate the suitability of different *Eucalyptus* tissue types as substrates for fungal colonization, samples exhibiting colonization by at least one endophytic fungus were enumerated. Colonization frequency (CF), also referred to as isolation rate, was calculated according to Equation 1 [47]. The CF was determined as the proportion of tissue sections colonized by endophytic fungi relative to the total number of incubated sections, following the methodology described in Equation 1 [48].


$$\begin{array}{c} CF\;(\%)=\;\frac{Number\;of\;plated\;samples}{Number\;of\;colonized\;samples}\times \text{1}\text{0}\text{0} \end{array}$$ (1)

Isolation frequency (IF) measures the occurrence of specific fungal species within a set of isolates. It is calculated by dividing the number of isolates of a particular species by the total number of isolates across all species, expressed as a percentage known as relative abundance (RA %). This calculation method is described by Huang et al. [48] and presented in Equation 2.


$$\begin{array}{c} RA\;(\%)\;=\frac{No.\;of\;isolates\;of\;a\;particular\;species}{Sum\;of\;isolates\;of\;all\;species}\;\times \;\text{1}\text{0}\text{0} \end{array}$$ (2)

The Chao1 estimator is considered a nonparametric approach for estimating species richness within a community. The method relies on the concept that rare species provide the most insight regarding the quantity of species that have not been detected. Specifically, the estimator targets species with low abundance by utilizing only singletons and doubletons, which correspond to species represented by one and two individuals, respectively, to infer the number of missing species. Therefore, datasets with a larger proportion of rare species tend to benefit the most from this index. The Chao1 richness estimate is calculated according to Equation 3, as introduced by Chao [49], where *S*_*obs*_ indicates the observed number of species, and *F*1 and *F*2 refer to the numbers of singletons and doubletons, respectively.


$$\begin{array}{c} S_{chao1}=S_{obs}\;+\;\frac{F\text{1}(F\text{1}\;-\;\text{1})}{\text{2}(F\text{2}\;+\;\text{1})} \end{array}$$ (3)

Species richness of distinct fungal isolates was calculated through the application of the Menhinick index, represented as *D*_*mn*_, using the formula outlined in Equation 4 and introduced by Whittaker [50]. In this expression, *S* stands for the number of unique fungal species observed per sample, while *N* designates the total number of fungal isolates recorded within the specific sample.


$$\begin{array}{c} D_{mn}\;=\;\frac{S}{\sqrt{\;}N} \end{array}$$ (4)

The Camargo evenness index is employed to evaluate the uniformity of species distribution within a community [51]. Calculation of this index is performed using Equation 5, in which *D*_*mn*_ denotes species richness.


$$\begin{array}{c} EC\;=\frac{\text{1}}{D_{mn}}\; \end{array}$$ (5)

The Shannon diversity index [52] is widely recognized as a fundamental metric for assessing diversity within fungal endophyte communities among various tissues. This index is calculated using Equation 6, where *pi* denotes the relative abundance of each species in a given sample. The value of the Shannon index, *H*′, ranges from zero, indicating no diversity when a single species is present, to higher values that signify increased diversity and a more equitable distribution of species. Thus, elevated values of *H*′ correspond to a greater uncertainty in predicting the species identity of an individual organism, which reflects a community with higher evenness.


$$\begin{array}{c} H^{\prime }\;=\;-\;\sum {\mathrm{\backslash nolimits}}_{i=\text{1}}^{s}pi\;ln\;pi \end{array}$$ (6)

Pielou’s evenness index, which is commonly employed to evaluate the uniformity of individual distribution among species within a community [53], was calculated according to Equation 7. In this equation, *J* represents Pielou’s measure of species evenness, *H*′ corresponds to the Shannon-Wiener diversity index for the sample, and *S* denotes the total number of species present within the sample.


$$\begin{array}{c} J\;=\;\frac{H^{\prime }}{ln\;(S)} \end{array}$$ (7)

Equation 8, introduced by Muthukrishnan et al. [54], was used to calculate the evenness index (*E*).


$$\begin{array}{c} E\;=\frac{e^{H^{\prime }}}{s} \end{array}$$ (8)

The Sorensen similarity index quantifies the ratio of twice the number of common species between two communities to the total number of species in both communities combined. This index was calculated using Equation 9 [55]. In this equation, *a* represents the number of fungal species common to both samples, while *b* and *c* denote the species unique to each respective sample.


$$\begin{array}{c} \beta_{Sor}\;=\frac{\text{2}c}{a\;+\;b\;+\;\text{2}c} \end{array}$$ (9)

The Jaccard similarity index measures the proportion of species common to two communities relative to the total species count in both communities combined. It is expressed using Equation 10 [56]. In this equation, *c* represents the number of species shared by both samples, while *a* and *b* denote the number of species unique to each sample, respectively. The index is calculated as the number of common species divided by the total number of species present in both communities combined, reflecting their similarity.


$$\begin{array}{c} \beta_{Jac}\;=\frac{c}{\left(a\;+\;b\;+\;c\right)} \end{array}$$ (10)

Fisher’s alpha serves as a parametric diversity index that is utilized to estimate species diversity under the assumption that species abundances follow a logarithmic distribution. The calculation of this index was conducted using Equation 11 [57]. Within this equation, *S* denotes the total number of species in the sample, *n* represents the total number of endophytic fungal isolates, and *α* (Fisher’s alpha) corresponds to the diversity parameter, which reflects the level of species diversity within the community. Greater values of Fisher’s alpha are indicative of higher species diversity.


$$\begin{array}{c} S=\;\alpha \;\times In\;(\text{1}\;+\;\frac{n}{\alpha }) \end{array}$$ (11)

The Berger-Parker dominance index measures the relative abundance of the most dominant species in a community, highlighting its numerical importance. The formula for the Berger-Parker index is given in Equation 12 [58], where *n*_*max*_ represents the number of individuals in the most abundant species, and *N* denotes the total number of individuals in the sample. The index is calculated by dividing *n*_*max*_ by *N*, reflecting the proportion of the dominant species within the community. The reciprocal of the Berger-Parker index (1/d) is commonly used, and an increase in this value indicates greater diversity and reduced dominance by the most abundant species.


$$\begin{array}{c} d\;=\frac{n_{max}}{N} \end{array}$$ (12)

The Simpson dominance index was applied to evaluate species diversity by quantifying the probability that two isolates randomly selected from a sample will belong to the same species (Equation 13) [59]. Within this equation, *ni* indicates the number of isolates of species *i*, and *N* represents the total number of isolates across all species. Additionally, the complement of the Simpson diversity index (1 − *D*) and the reciprocal of the Simpson index (1/*D*) were also calculated to provide alternative measures of diversity.


$$\begin{array}{c} D\;=\frac{\sum_{\text{1}}^{s}ni\;(ni\;s\;-\;\text{1})\;}{N\;(N\;-\;\text{1})} \end{array}$$ (13)

The complement of the Simpson diversity index, expressed as 1 − *D*, and the reciprocal of the Simpson index, expressed as 1/*D*, were additionally calculated to provide further measures of species diversity and evenness.

Species richness was evaluated by using the Margalef index, as expressed in Equation 14 [60]. Within this formula, *S* denotes the total number of species observed, while *N* represents the total number of isolates counted.


$$\begin{array}{c} D_{Mg}=\;\frac{S\;-\;\text{1}}{ln(N)} \end{array}$$ (14)

Data analysis and graph generation were performed using Microsoft Excel (2021) and the Python programming language with the aid of several libraries including matplotlib, seaborn, plotly, and matplotlib_venn. Additionally, pandas and numpy were employed for data manipulation and scipy for statistical modeling. These tools facilitated the production of heatmaps, stacked bar charts, Sankey diagrams, Venn diagrams, and other graphical representations essential for analysis and presentation.

## Results

### Endophytic fungal isolates

No fungal growth was observed on WA plates in the control group, which confirmed the effectiveness of the surface sterilization process. As a result, all fungi isolated in this study can be regarded as endophytes, since pure single clones were successfully obtained from each tissue. A total of 712 endophytic fungal isolates were obtained from 660 cultured tissue segments of *E. camaldulensis*, which were collected from Tehran (17 samples), Qom (7 samples), Alborz (4 samples), Isfahan (6 samples), and Mazandaran (11 samples) provinces. These isolates were classified into two phyla, six classes, seventeen orders, and twenty-two families. Following a detailed examination of the characteristics and growth patterns of the colonies, forty-two distinct morphotypes were identified, reflecting the diversity among the total isolates.

**Fig 1:**
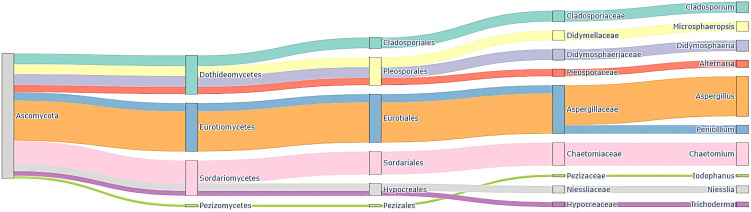
Taxonomic Relationships of Species: the endophytic fungi isolated from *Eucalyptus camaldulensis* in Qom province belonged to 1 phylum, 4 classes, 6 orders, 9 families, and 10 genera.

**Fig 2:**
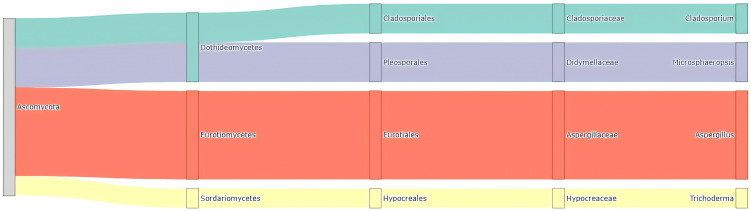
Taxonomic Relationships of Species: the endophytic fungi isolated from *Eucalyptus camaldulensis* in Alborz province belonged to 1 phylum, 3 classes, 4 orders, 4 families, and 4 genera.

**Fig 3:**
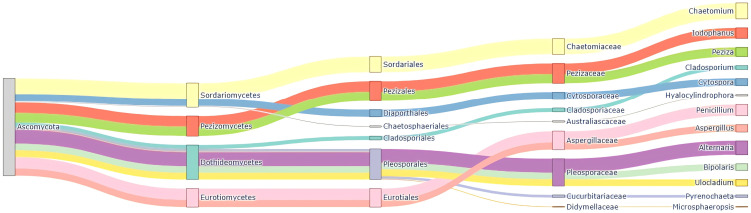
Taxonomic Relationships of Species: the endophytic fungi isolated from *Eucalyptus camaldulensis* in Tehran province belonged to 1 phylum, 4 classes, 7 orders, 9 families, and 13 genera.

**Fig 4:**
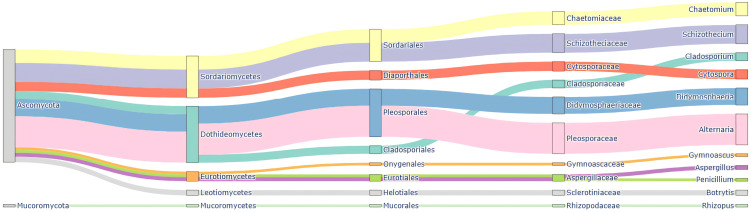
Taxonomic Relationships of Species: the endophytic fungi isolated from *Eucalyptus camaldulensis* in Isfahan province belonged to 2 phyla, 5 classes, 8 orders, 10 families, and 11 genera.

**Fig 5:**
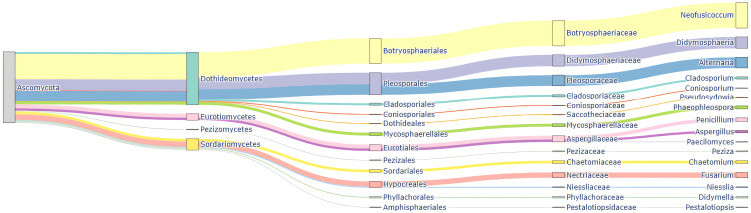
Taxonomic Relationships of Species: the endophytic fungi isolated from *Eucalyptus camaldulensis* in Mazandaran province belonged to 1 phylum, 4 classes, 12 orders, 14 families, and 16 genera.

Morphological and molecular characterization of endophytic fungi isolated from *E. camaldulensis* across five provinces revealed a predominance of the phylum Ascomycota, with a minor presence of Mucoromycota in Isfahan. In Qom, 103 isolates were Ascomycota, distributed among four classes including Dothideomycetes (31.07%), Eurotiomycetes (38.83%), Sordariomycetes (28.16%), and Pezizomycetes (1.94%) spanning 12 orders, nine families, and 10 genera. Alborz yielded 18 isolates, all Ascomycota, across three classes (Sordariomycetes, Dothideomycetes, Eurotiomycetes), four orders, four families, and four genera. Tehran had 147 isolates across four classes (Dothideomycetes, Eurotiomycetes, Sordariomycetes, Pezizomycetes), seven orders, nine families, and 13 genera. Isfahan had 86 isolates, with 84 isolates (98.78%) in Ascomycota distributed among Dothideomycetes, Sordariomycetes, Eurotiomycetes, and Leotiomycetes and two isolates (1.22%) in Mucoromycota (Mucoromycetes), collectively spanning five classes, eight orders, 10 families, and 11 genera. Mazandaran showed the highest diversity with 358 isolates, all Ascomycota, across four classes (Dothideomycetes, Sordariomycetes, Eurotiomycetes, Pezizomycetes), 13 orders, 15 families, and 16 genera; Dothideomycetes dominated (74.30%), followed by Sordariomycetes (15.64%), Eurotiomycetes (8.94%), and Pezizomycetes (1.12%) (Figs 1–5).

**Fig 6:**
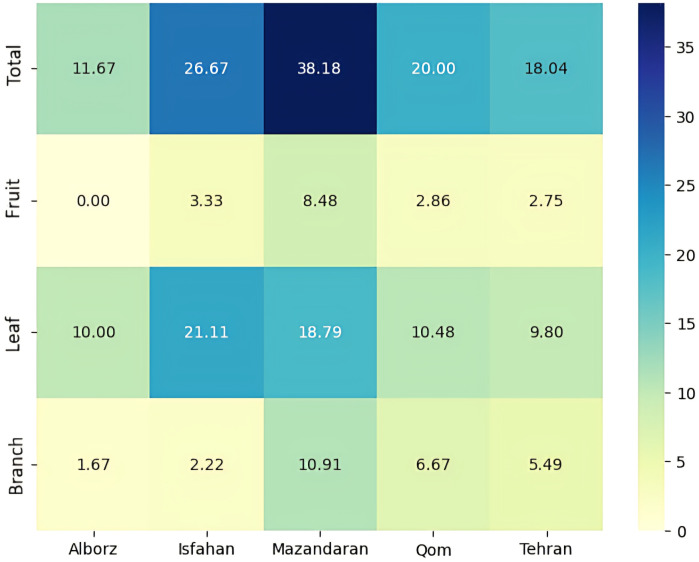
Colonization Frequency (CF) of endophytic fungi in different tissues across five provinces of Iran. The heatmap shows the CF (%) in leaf, branch, fruit, and total tissue for each province. Darker colors indicate higher colonization rates, highlighting tissue-specific and province-specific patterns of fungal colonization.

The colonization frequency (CF) analysis highlighted provincial and tissue-specific patterns. Mazandaran exhibited the highest overall CF (38.18%), followed by Isfahan (26.67%), Qom (20.00%), Tehran (18.04%), and Alborz (11.67%). Across all provinces, leaf tissues consistently showed the highest CF values (e.g., Mazandaran: 18.79%, Isfahan: 21.11%), branches intermediate, and fruits the lowest, indicating leaves as the primary niche for endophytic fungi (Fig 6).

**Fig 7:**
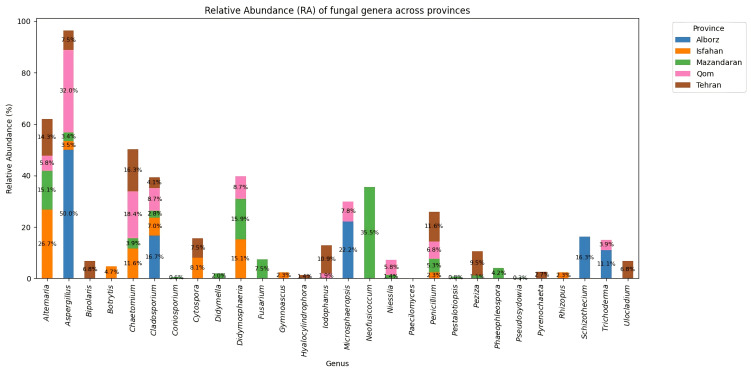
Relative Abundance (RA) of fungal genera across five provinces of Iran. Each stacked bar represents a fungal genus, and the colored segments indicate the percentage contribution of each province to that genus. Numbers on the bars show exact RA values (%), facilitating comparison of fungal community composition among provinces.

Relative abundance (RA%) analysis revealed distinct fungal community structures. In Alborz, genus *Aspergillus* (50%) and *Microsphaeropsis* (22.2%) dominated, reflecting low diversity. Isfahan exhibited higher diversity with *Alternaria* (26.74%), *Didymosphaeria* (15.12%), and *Schizothecium* (16.28%) as major genera. Mazandaran had the richest and most balanced community, dominated by *Neofusicoccum* (35.47%) and *Didymosphaeria* (15.92%), consistent with its highest CF. Qom was dominated by *Chaetomium* (18.45%) and *Cladosporium* (8.74%), whereas Tehran showed moderate diversity with *Alternaria* (14.29%), *Chaetomium* (16.33%), *Penicillium* (11.56%), and *Iodophanus* (10.88%) (Fig 7).

Integration of CF and RA% data indicates that provinces with higher colonization, such as Mazandaran and Isfahan, also possess greater genus richness and more evenly distributed communities. Conversely, lower CF provinces, like Alborz, are dominated by few genera, reflecting restricted community composition. Leaf tissues act as primary reservoirs of fungal diversity, while branch and fruit tissues harbor fewer isolates and are more genus-specific, suggesting niche specialization. Certain genera display provincial specificity, such as *Neofusicoccum* in Mazandaran or *Schizothecium* in Isfahan, reflecting local environmental conditions and tissue preferences. Collectively, these results demonstrate province- and tissue-specific structuring of endophytic fungal communities, where colonization intensity (CF) and community composition (RA%) are closely linked. Leaf tissues consistently support the highest colonization and diversity, whereas branches and fruits contribute selectively to genus-specific assemblages, illustrating habitat preference and regional variability in fungal endophyte distribution.

A total of 28 fungal genera were isolated across the provinces of Isfahan, Tehran, Qom, Alborz, and Mazandaran, showing distinct patterns of distribution. Among these, *Cladosporium* and *Aspergillus* were ubiquitous, being detected in all five provinces. Genera with widespread distribution included *Chaetomium* and *Alternaria*, each present in four provinces, indicating a broad ecological adaptability. Some genera exhibited moderate distribution, being isolated in three provinces: *Microsphaeropsis* (Tehran, Qom, Alborz) and *Didymosphaeria* (Isfahan, Qom, Mazandaran), suggesting localized abundance patterns. Several genera were restricted to two provinces, including *Cytospora* (Isfahan, Tehran), *Iodophanus* (Tehran, Qom), *Peziza* (Tehran, Mazandaran), *Trichoderma* (Qom, Alborz), and *Niesslia* (Qom, Mazandaran), reflecting partial regional specificity. A number of genera were highly localized, occurring in only a single province. These include *Schizothecium*, *Gymnoascus*, *Botrytis* in Isfahan; *Pyrenochaeta*, *Hyalocylindrophora*, *Bipolaris*, *Ulocladium* in Tehran; and *Neofusicoccum*, *Coniosporium*, *Pseudosydowia*, *Phaeophleospora*, *Paecilomyces*, *Fusarium*, *Didymella*, *Pestalotiopsis* in Mazandaran.

### Alpha diversity assessment of endophytic fungi in *E. camaldulensis* across Alborz, Isfahan, Mazandaran, Qom, and Tehran provinces

**Fig 8:**
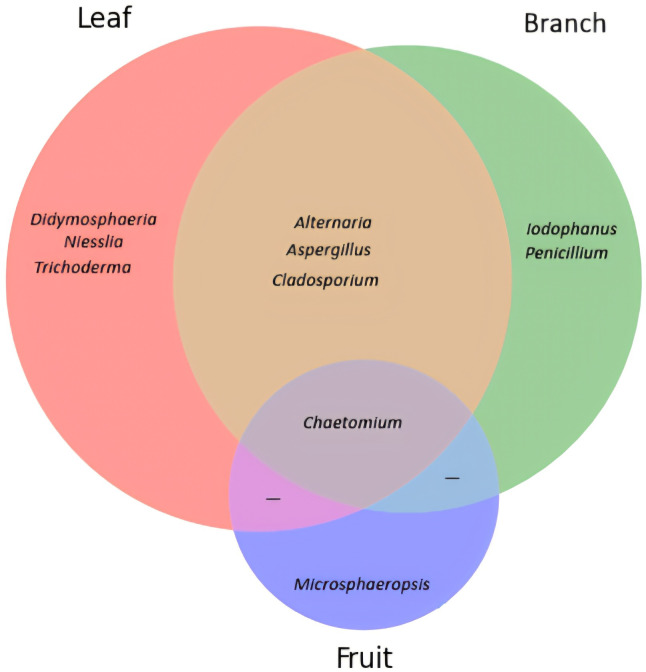
Venn diagram of endophytic fungal species isolated from leaves, branches and fruits of *Eucalyptus camaldulensis* grown in Qom province.

**Fig 9:**
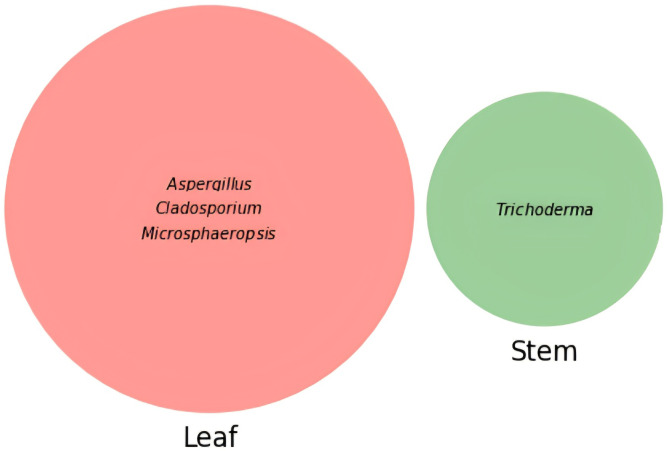
Venn diagram of endophytic fungal species isolated from leaves and branches of *Eucalyptus camaldulensis* grown in Alborz province.

**Fig 10:**
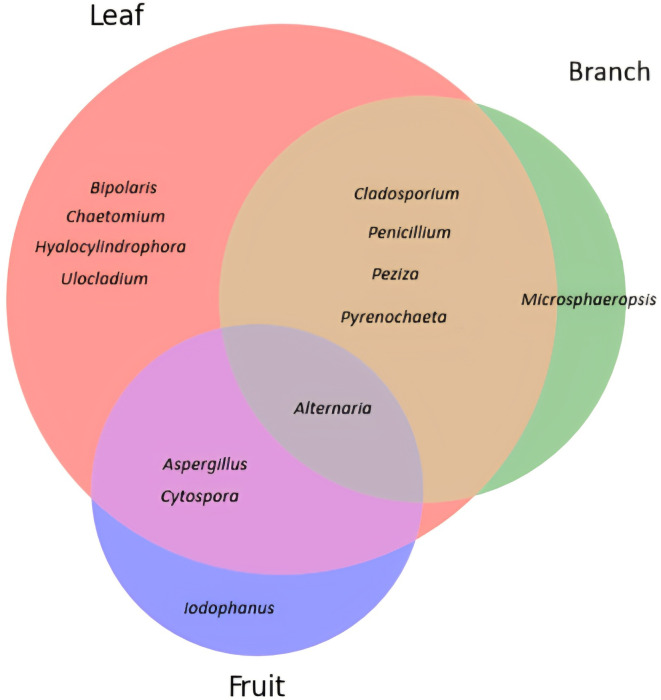
Venn diagram of endophytic fungal species isolated from leaves and branches of *Eucalyptus camaldulensis* grown in Tehran province.

**Fig 11:**
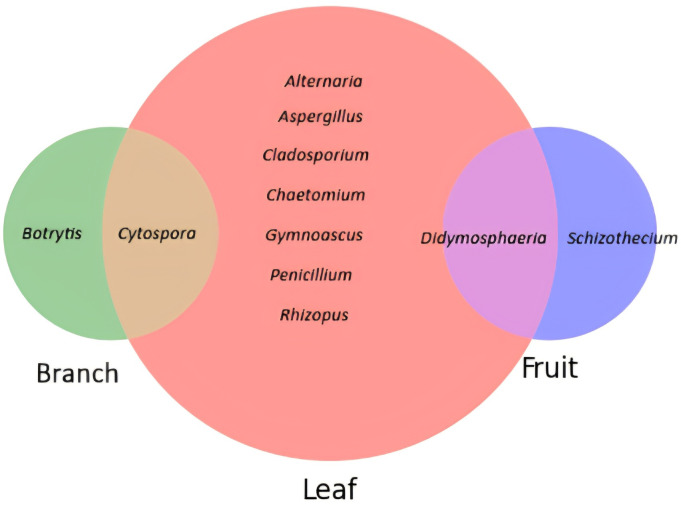
Venn diagram of endophytic fungal species isolated from leaves and branches of *Eucalyptus camaldulensis* grown in Isfahan province.

**Fig 12:**
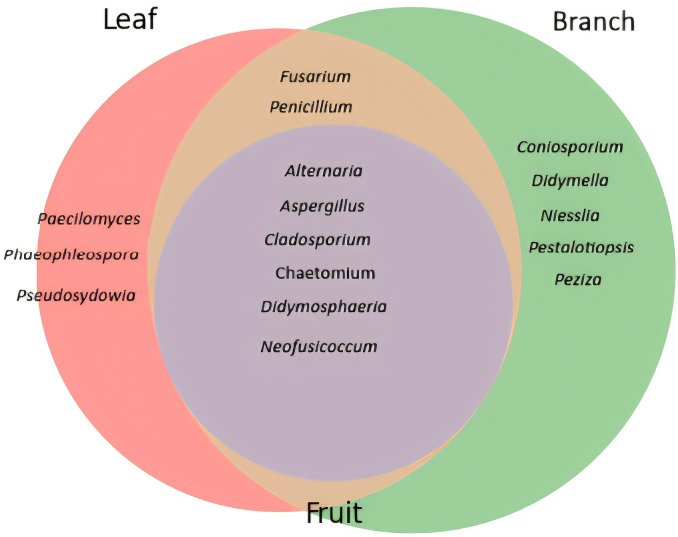
Venn diagram of endophytic fungal species isolated from leaves and branches of *Eucalyptus camaldulensis* grown in Mazandaran province.

The fungal species identified in tissue samples from each province are shown in Figs 8–12. The Shannon-Wiener index (H) was used for quantifying species diversity within a community, incorporating both species richness and the evenness of their abundances. When only one species is present, the index equals zero. Higher values indicate increased diversity, particularly in communities where individuals are distributed more evenly across multiple species [61].

The Shannon–Wiener index (H) and Simpson dominance index (D) revealed that the endophytic fungal communities associated with *E. camaldulensis* in different provinces exhibited varying levels of diversity depending on plant tissue type. The highest diversity was observed in leaf tissue from Mazandaran (H = 1.955, D = 0.172), followed by leaf tissue from Isfahan (H = 1.858, D = 0.199). In contrast, the lowest diversity was recorded in fruit tissue from Isfahan (H = 0.245, D = 0.876), and branch tissue from Alborz (H = 0.000, D = 1.000), where no variation was detected. Notably, no endophytic fungi were isolated from fruit tissue in Alborz province. Among all tissue types, fruit generally showed the lowest diversity, while leaf tissues harbored more diverse fungal communities across the provinces. These findings emphasize that endophytic fungal diversity in *E. camaldulensis* is strongly influenced by both plant tissue type and geographic region (Table 1).

**Table 1:** Chao richness estimator, Margalef diversity index, Shannon-Wiener diversity index, Simpson dominance index (D), Simpson diversity index (1-D), and reciprocal of Simpson index (1/D) calculated for the endophytic fungal species community isolated from leaves, branches, and fruits of *E. camaldulensis* collected from Alborz, Isfahan, Mazandaran, Qom, and Tehran provinces.

Province	Tissue	Chao richness estimator	Margalef diversity index	Shannon-Wiener diversity index	Simpson dominance index (D)	Simpson diversity index (1-D)	Reciprocal of Simpson index (1/D)
Alborz	total	4	1.038	1.224	0.34	0.66	2.945
Alborz	leaf	3	0.721	0.984	0.414	0.586	2.415
Alborz	branch	1	0	0	1	0	1
Isfahan	total	11	2.245	2.096	0.151	0.849	6.627
Isfahan	fruit	2	0.369	0.245	0.876	0.124	1.142
Isfahan	leaf	9	1.909	1.858	0.199	0.801	5.03
Isfahan	branch	2	0.621	0.5	0.68	0.32	1.471
Mazandaran	total	18	2.551	2.058	0.188	0.812	5.306
Mazandaran	fruit	6	1.029	1.351	0.314	0.686	3.19
Mazandaran	leaf	13	1.996	1.955	0.172	0.828	5.814
Mazandaran	branch	13	2.746	2.199	0.166	0.834	6.007
Qom	total	12	2.373	2.259	0.124	0.876	8.055
Qom	fruit	2	0.402	0.637	0.556	0.444	1.8
Qom	leaf	7	1.454	1.726	0.212	0.788	4.722
Qom	branch	6	1.485	1.588	0.241	0.759	4.143
Tehran	total	13.5	2.405	2.363	0.104	0.896	9.574
Tehran	fruit	4	0.932	1.001	0.462	0.538	2.163
Tehran	leaf	11	2.222	2.187	0.135	0.865	7.391
Tehran	branch	6.25	1.443	1.514	0.252	0.748	3.969

**Fig 13:**
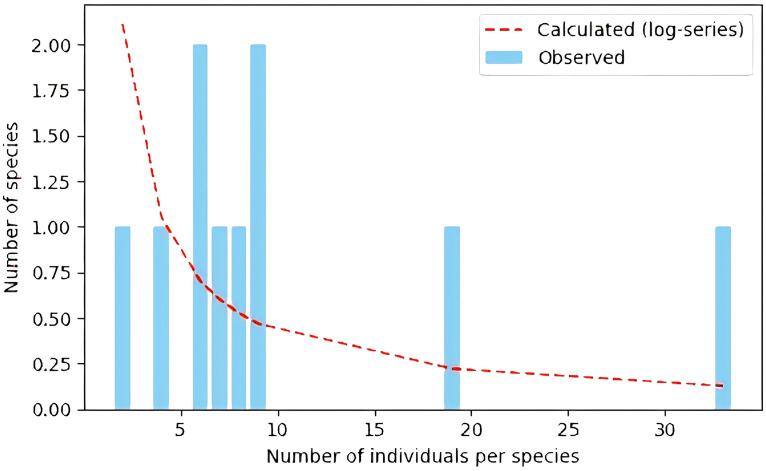
Species abundance distribution for endophytic fungal isolates obtained from the leaves, branches, and fruits of the *Eucalyptus camaldulensis* from Qom.

**Fig 14:**
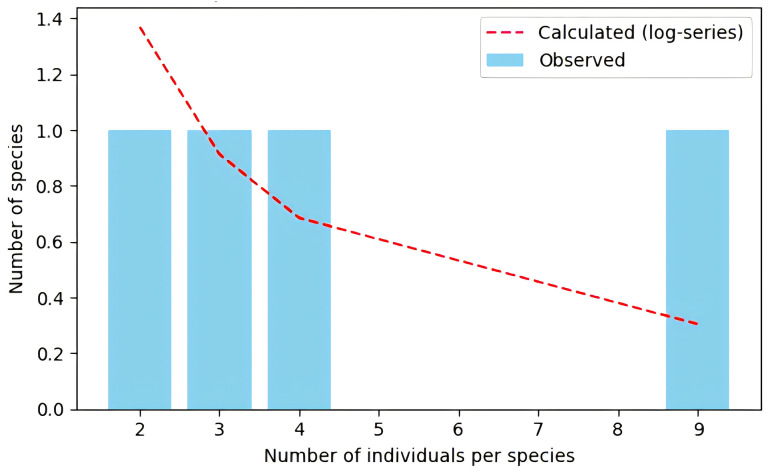
Species abundance distribution for endophytic fungal isolates obtained from the leaves, branches, and fruits of the *Eucalyptus camaldulensis* from Alborz.

**Fig 15:**
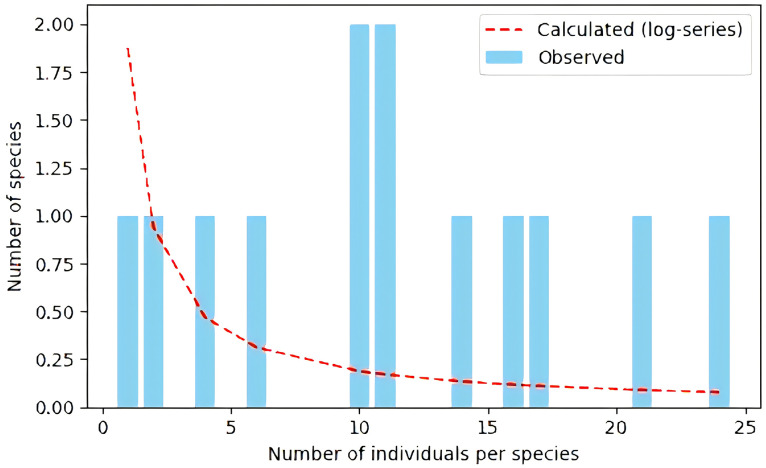
Species abundance distribution for endophytic fungal isolates obtained from the leaves, branches, and fruits of the *Eucalyptus camaldulensis* from Tehran.

**Fig 16:**
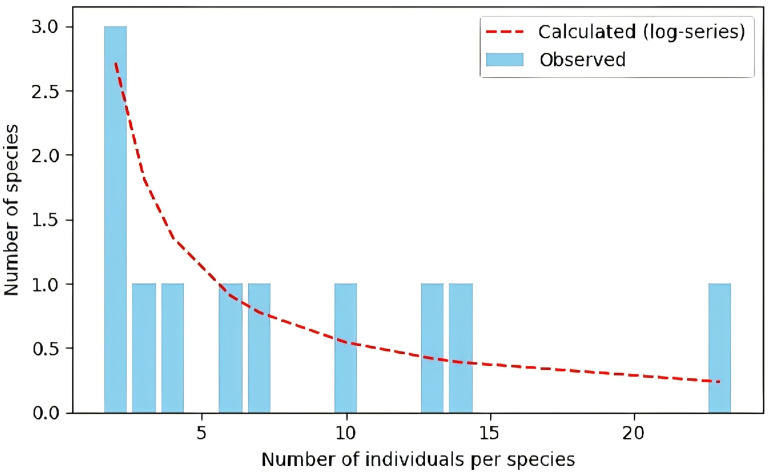
Species abundance distribution for endophytic fungal isolates obtained from the leaves, branches, and fruits of the *Eucalyptus camaldulensis* from Isfahan.

**Fig 17:**
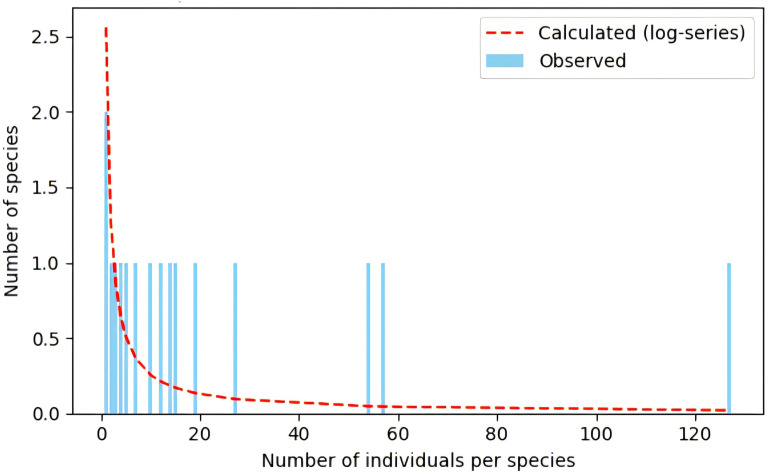
Species abundance distribution for endophytic fungal isolates obtained from the leaves, branches, and fruits of the *Eucalyptus camaldulensis* from Mazandaran.

Understanding the relationship between abundance and diversity in endophytic fungal communities is essential for interpreting ecological dynamics within host plants. In alignment with these findings, the frequency distribution of endophytic fungal species isolated from Alborz, Isfahan, Qom, Tehran, and Mazandaran provinces follows a Poisson distribution (Figs 13–17), suggesting a random colonization pattern. The relatively high diversity and low dominance indices observed in Mazandaran leaf and branch tissues (H = 1.955–2.199; D = 0.166–0.172) support the possibility of a similar distribution pattern in this region.

Fisher’s alpha diversity index was used to evaluate the species richness of endophytic fungi isolated from different tissues of *E*. *camaldulensis* across five provinces (Table 2). The results showed considerable variation depending on both tissue type and geographic location. Branch and leaf tissues generally exhibited higher alpha diversity than fruit tissues. The highest alpha value was recorded in branch tissues from Mazandaran (α = 4.428), followed by leaf tissues from Tehran (α = 3.288) and Isfahan (α = 2.816), suggesting high species richness and evenness in these regions. In contrast, fruit tissues exhibited the lowest alpha values, particularly in Qom (α = 0.685) and Isfahan (α = 0.620), indicative of low diversity and potential dominance by a few taxa. Alborz province showed generally low diversity as well, with values of α = 1.090 in leaf and α = 0.796 in branch tissues, and no fungal isolates were recovered from its fruit tissue. These findings underscore the role of both host tissue specificity and regional ecological factors in shaping the diversity of endophytic fungal communities in *E. camaldulensis* (Figs 13–17).

**Table 2:** Berger–Parker dominance index, reciprocal of Berger–Parker index, Pielou evenness index, Menhinick index, Camargo index, and Fisher alpha calculated for the endophytic fungal species community isolated from leaves, branches, and fruits of *E. camaldulensis* collected from Alborz, Isfahan, Mazandaran, Qom, and Tehran provinces.

Province	Tissue	Berger–Parker dominance index	Reciprocal of Berger–Parker index	Pielou Evenness index	Menhinick index	Camargo index	Evenness (e^H′)/S	Fisher_alpha
Alborz	total	0.5	2	0.883	0.943	0.796	0.85	1.594
Alborz	leaf	0.562	1.778	0.896	0.75	0.75	0.892	1.09
Alborz	branch	1	1		0.707	–	1	0.796
Isfahan	total	0.267	3.739	0.874	1.186	0.914	0.74	3.35
Isfahan	fruit	0.933	1.071	0.353	0.516	0.133	0.639	0.62
Isfahan	leaf	0.348	2.87	0.845	1.108	0.888	0.712	2.816
Isfahan	branch	0.8	1.25	0.722	0.894	0.4	0.825	1.235
Mazandaran	total	0.355	2.819	0.742	0.846	0.917	0.49	3.437
Mazandaran	fruit	0.45	2.224	0.754	0.528	0.798	0.644	1.303
Mazandaran	leaf	0.273	3.659	0.815	0.898	0.898	0.642	2.734
Mazandaran	branch	0.354	2.821	0.857	1.463	0.931	0.694	4.428
Qom	total	0.214	4.682	0.909	1.182	0.933	0.797	3.519
Qom	fruit	0.667	1.5	0.918	0.577	0.667	0.945	0.685
Qom	leaf	0.355	2.818	0.887	0.889	0.879	0.802	2.028
Qom	branch	0.379	2.636	0.886	1.114	0.86	0.815	2.297
Tehran	total	0.163	6.125	0.921	1.072	0.943	0.817	3.441
Tehran	fruit	0.64	1.562	0.722	0.8	0.7	0.68	1.344
Tehran	leaf	0.267	3.75	0.912	1.16	0.933	0.81	3.288
Tehran	branch	0.312	3.2	0.845	1.061	0.846	0.758	2.18

### Beta diversity analysis of endophytic fungi in different *Eucalyptus* tissues across five provinces

Beta diversity, introduced by Whittaker [62], measures the variation in species composition between ecosystems within a region. Two common nonparametric indices for this are the Sorensen (βSor) [55] and Jaccard (βJac) [56] indices. Both compare species presence between communities, but the Sorensen index gives more weight to shared species, while the Jaccard index treats all species equally.

Beta diversity analysis combining Jaccard and Sørensen indices revealed distinct tissue-specific patterns and notable regional contrasts across the studied provinces. Within provinces, Qom showed the highest leaf–branch similarity (J = 0.444; S = 0.833), whereas fruit–leaf (J = 0.125; S = 0.364) and fruit–branch (J = 0.143; S = 0.364) similarities were moderate, indicating partial sharing of genera with fruit tissues. In Tehran, leaf– branch similarity was moderate (J = 0.417; S = 0.667), while fruit–leaf (J = 0.25; S = 0.429) and fruit–branch (J = 0.111; S = 0.182) overlaps were lower, reflecting limited genus sharing with fruits. Mazandaran exhibited high similarity across all tissue types—leaf– branch (J = 0.500; S = 0.625), fruit–leaf (J = 0.545; S = 0.588), and fruit– branch (J = 0.462; S = 0.471), suggesting a broader genus pool shared among tissues. Isfahan displayed low cross-tissue similarity, with leaf– branch (J = 0.200; S = 0.333) and minimal fruit–leaf (J = 0.100; S = 0) or fruit– branch (J = 0.250; S = 0) overlap, whereas Alborz showed complete tissue compartmentalization, with zero leaf– branch overlap (J = 0; S = 0) and no fruit data available.

Between provinces for the same tissue, leaf communities were most similar between Isfahan and Qom (J = 0.454; S = 0.571), and between Isfahan and Mazandaran (J = 0.429; S = 0.462), while Alborz showed the lowest similarity with Mazandaran (J = 0.167; S = 0.211) and Isfahan (J = 0.200; S = 0.267). Branch communities were most similar between Mazandaran and Qom (J = 0.357; S = 0.571) and Qom–Tehran (J = 0.333; S = 0.636), with several province pairs, particularly involving Alborz, sharing no branch genera (J = 0; S = 0). Fruit communities displayed highest similarity between Isfahan and Mazandaran (J = 0.143; S = 0.400), and Qom–Tehran (J = 0; S = 0.400), whereas other province pairs had minimal or zero similarity, indicating strong regional turnover.

Overall, the analyses indicate that fungal community composition is strongly influenced by tissue type and regional context. Fruit tissues consistently harbor distinctive assemblages with limited sharing, whereas leaf and branch communities exhibit higher similarity within and between certain provinces, particularly Qom, Mazandaran, and Tehran. Alborz stands out as compositionally distinct, highlighting pronounced regional turnover. The combined use of Jaccard and Sørensen indices demonstrates that both presence–absence patterns and relative abundance contribute to the observed variation, emphasizing that endophytic fungal communities are structured by a complex interplay of tissue specificity and geographic differentiation.

### Richness of the species

Mazandaran displayed the highest species richness (S = 16), followed by Tehran (S = 13), Qom (S = 12), Isfahan (S = 11), and Alborz (S = 4). Regarding fungal abundance, leaf tissues consistently yielded the highest number of isolates across all provinces, with lower numbers recovered from branch and fruit tissues. This pattern suggests that leaf tissues serve as a more favorable niche for endophytic fungi colonization in *E*. *camaldulensis*.

Since species count (S) is influenced by sampling effort, it does not provide a standardized basis for comparing diversity across regions or tissue types. Therefore, the Margalef and Menhinick indices were applied to estimate species richness independently of sample size. Among the five provinces, Mazandaran consistently exhibited the highest richness values, particularly in leaf and branch tissues. Tehran and Qom showed moderate richness, whereas Isfahan and Alborz had the lowest, especially in branch and fruit tissues.

The Berger–Parker dominance index identified high dominance levels in the fruit tissues of Isfahan (0.933), as well as in the branch tissue of Alborz (1.000), indicating that a single genus accounted for most of the fungal isolates in these compartments. A relatively high dominance was also observed in the fruit tissue of Qom (0.667), though to a lesser extent. In contrast, lower dominance values were recorded in the leaf tissues of Tehran (0.267) and Mazandaran (0.273), reflecting greater species evenness and diversity. These patterns collectively highlight the dual influence of tissue compartment and geographic location on the richness and structure of endophytic fungal communities in *E*. *camaldulensis*.

According to the Chao1 richness estimator, notable variation in estimated species richness of endophytic fungi was observed among the studied provinces. Mazandaran exhibited the highest overall richness (8.25), suggesting a comparatively diverse fungal community, while Alborz recorded the lowest value (2.75), indicating a limited number of taxa. In all provinces, leaf tissues consistently harbored higher estimated richness compared to branches and fruits, highlighting a recurring pattern across different environmental contexts. At the tissue level, the highest Chao1 value was recorded in the leaves of Mazandaran (5.00), whereas the lowest was found in the branches of Alborz (0.50) (Table 1).

### Species evenness

Species evenness, or equitability, is a key component of biodiversity assessments, as it reflects how uniformly individuals are distributed across different fungal taxa within a community. In studies of endophytic fungi, evenness helps to clarify whether a community is dominated by a few species or if most species contribute similarly to the overall population. When all species are equally abundant, evenness reaches its maximum value of 1; as the distribution becomes more uneven, the index decreases toward 0 [63].

Among the studied provinces, Qom exhibited the highest overall evenness, with a Pielou index of 0.913, indicating a relatively uniform distribution of fungal isolates among different genera. Tehran also showed a similarly high level of equitability (0.911). Despite its relatively low number of total isolates, Alborz ranked third (0.883), reflecting a balanced community structure. Isfahan (0.846) and Mazandaran (0.840) followed closely, suggesting moderately even fungal distributions. When plant tissues were considered, leaf samples generally demonstrated higher equitability compared to other tissues. For instance, leaf tissues in Tehran (0.912), Qom (0.887), and Alborz (0.896) supported well-balanced fungal communities. In contrast, extremely low evenness was recorded in the branch tissues of Alborz (0.000), indicating the dominance of a single genus. Similarly, fruit tissues from Isfahan displayed a relatively low value (0.353), suggesting an uneven distribution of taxa. These patterns highlight considerable spatial and tissue-specific variation in the distribution of endophytic fungi. Consistently, leaf tissues harbored more balanced communities, whereas branches—particularly in certain regions—tended to be dominated by a single taxon. The Camargo Uniformity Index corroborated these results, showing strong agreement with both Pielou’s evenness and Berger–Parker dominance metrics (Table 2). Collectively, these indices emphasize that leaf tissues generally sustain more equitable fungal communities than branches, while fruits display intermediate levels of evenness depending on the geographic region.

The Simpson’s Dominance Index (D) ranges from 0 to 1, where values approaching zero indicate high species diversity and community stability, reflecting the absence of dominance by any single species. Conversely, values nearing one suggest low diversity, dominance by one or a few species, and potential ecosystem instability due to ecological pressures [64].

In the present study, the D values for most tissues across all provinces were relatively low, signifying a stable and diverse endophytic fungal community structure. This pattern was particularly evident in leaf and branch tissues, with Tehran leaf (D = 0.104), Mazandaran branch (D = 0.166), and Mazandaran leaf (D = 0.172) exhibiting the highest diversity and evenness among the sampled tissues. Notable exceptions were observed in the fruit tissues of Qom (D = 0.556) and Tehran (D = 0.462), as well as the branch tissue of Alborz (D = 1.000) and fruit tissue of Isfahan (D = 0.876). These elevated D values indicate the dominance of one or a few fungal species, which may reflect ecological constraints or limited colonization niches within these specific tissues. Overall, these findings suggest that the endophytic fungal community structure is generally stable and diverse across most provinces and tissue types, except for reduced diversity in fruit tissues of Qom and Tehran, and branch and fruit tissues of Alborz and Isfahan, respectively (Table 1). The Simpson’s Diversity Index (1–D), which quantifies species diversity within a community, corroborates these observations; higher values closer to one represent greater diversity, whereas lower values indicate diminished diversity [46,65]. As shown in Table 1, the 1–D values align with the dominance index results, confirming that most tissues harbor diverse and evenly distributed endophytic fungal populations, except in the aforementioned tissues where species dominance is apparent.

**Fig 18:**
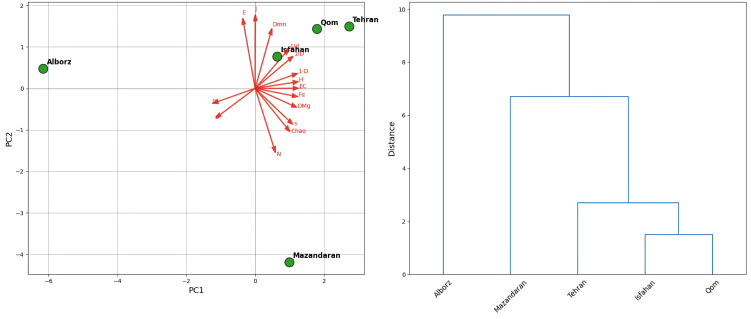
Principal component analysis (A) and classification dendrogram (B) based on calculated diversity indices for fungal species from Qom, Alborz, Tehran, Isfahan and Mazandaran provinces. Chao, Chao richness estimator; DMg, Margalef diversity index; H′, Shannon–Wiener diversity index; D, Simpson dominance index; 1-D, Simpson diversity index; 1/D, Reciprocal of Simpson index; d, Berger Parker dominance index; 1/d, Reciprocal of Berger–Parker index; J, Pielou evenness index; Dmn, Menhinick index; EC, Camargo index; E, Evenness_(e^H′)/S; Fisher alpha.

Principal Component Analysis (PCA) was conducted to investigate patterns in the endophytic fungal community structure across different plant tissues (leaf, branch, and fruit) from the provinces of Alborz, Isfahan, Tehran, Qom, and Mazandaran. Five ecological indices were used in the analysis: Margalef and Menhinick richness indices, Berger-Parker dominance index, Camargo’s evenness index, and Simpson’s dominance index (D). The first principal component (PC1) accounted for the largest proportion of variance and was predominantly associated with dominance-related indices, namely Berger-Parker, Simpson’s D, and Camargo’s evenness. These indices represent species dominance and community uniformity, where higher values along this axis correspond to communities dominated by a few species and consequently lower overall diversity. The second principal component (PC2) explained a smaller fraction of the variance and was mainly influenced by richness indices (Margalef and Menhinick), which reflect the number of taxa relative to sample size and are directly linked to species diversity. Samples such as branch tissues from Mazandaran and leaf tissues from Tehran, characterized by high richness and low dominance, were positioned toward the richness-dominated end of the PCA space, indicating more diverse and evenly distributed fungal communities. Conversely, samples such as fruit tissue from Isfahan and branch tissue from Alborz, with the highest dominance and lowest richness values, were located toward the opposite extreme, suggesting communities with low diversity dominated by one or a few species. Overall, PC1 can be interpreted as a gradient of species dominance, while PC2 represents species richness. This analysis effectively highlights the contrasting community structures of endophytic fungi across different provinces and tissue types. According to Fig 18, provinces like Tehran and Mazandaran exhibit higher species diversity and more stable community structures, particularly in leaf and branch tissues, whereas other provinces and tissue types tend to display more uneven communities dominated by specific taxa.

Based on the calculated diversity indices, a dendrogram was constructed (Fig 18) to evaluate the similarity of endophytic fungal communities among the five studied provinces. The results demonstrated that Qom and Tehran clustered closely together, exhibiting the greatest similarity at a relatively low dissimilarity threshold. Isfahan joined this cluster at a moderately higher dissimilarity level, reflecting an intermediate degree of similarity. In contrast, Alborz and Mazandaran formed distinct branches at higher dissimilarity levels, indicating less similarity to the other provinces and to each other. This hierarchical clustering pattern suggests that the fungal communities in Qom and Tehran share comparable diversity and dominance characteristics, whereas Alborz and Mazandaran harbor more distinct community compositions. Overall, this clustering provides valuable insights into the ecological variation and biogeographical distribution of endophytic fungi across the different studied regions.

### Community structuring and categorization

**Fig 19:**
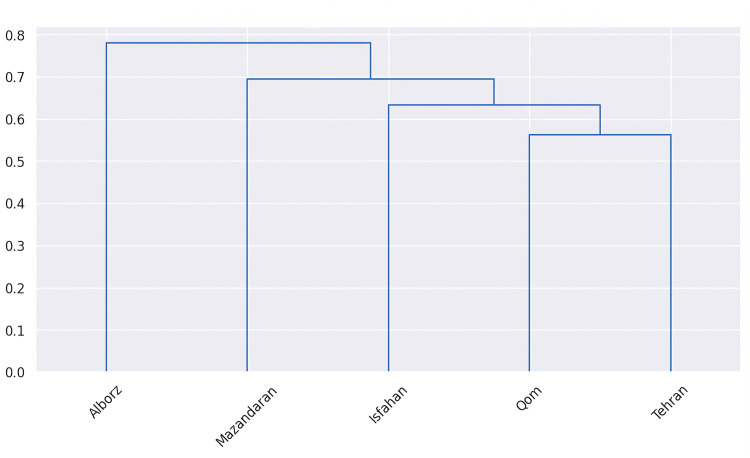
Dendrogram of the classification of fungal species from Alborz, Qom, Tehran, Isfahan, and Mazandaran provinces based on Jaccard similarity.

Classifying species is essential for understanding complex ecosystems. To identify the natural factors that drive species to form distinct groups, community classification methods are a crucial tool [66]. In this study, cluster analysis was performed using the Jaccard similarity index to evaluate the compositional similarity of endophytic fungal communities among the provinces of Qom, Alborz, Tehran, Isfahan, and Mazandaran. The resulting dendrogram (Fig 19) revealed that Tehran and Qom shared the highest similarity in fungal genera composition, forming a cluster at the lowest dissimilarity level. This primary cluster was subsequently joined by Alborz at a slightly higher dissimilarity level. In contrast, Isfahan and Mazandaran formed a separate cluster, reflecting their more distinct fungal communities. Overall, the pattern demonstrates that Tehran and Qom are the most similar pair, with Alborz closely related, while Isfahan and Mazandaran exhibit greater compositional distinction.

### Specificity of community composition

**Fig 20:**
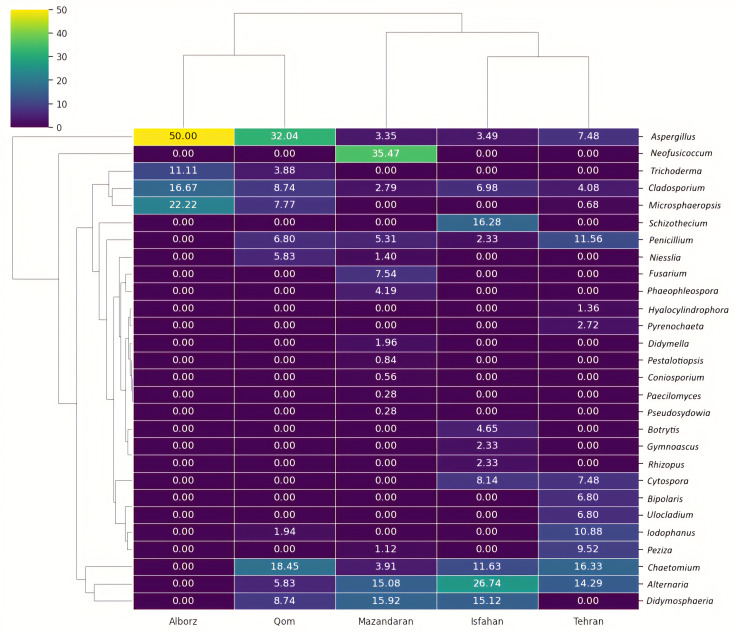
Heatmap shows the relative frequency of endophytic fungal genera isolated from *Eucalyptus camaldulensis* in Alborz, Qom, Tehran, Isfahan and Mazandaran provinces.

**Fig 21:**
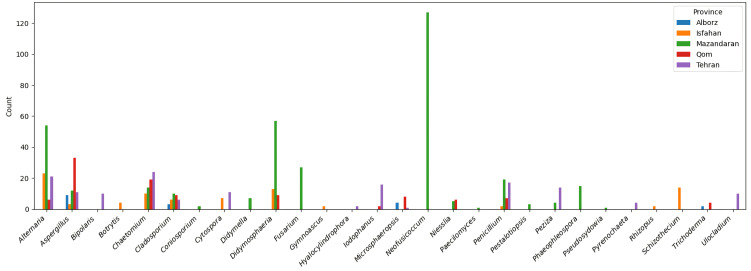
The relative abundance of endophytic fungal genera isolated from *Eucalyptus camaldulensis* in Alborz, Qom, Tehran, Isfahan and Mazandaran provinces.

Among the studied endophytic fungi, several common genera including *Cladosporium*, *Chaetomium*, *Alternaria*, *Aspergillus*, and *Penicillium*, were found across all five provinces. Among these, *Aspergillus* and *Alternaria* were generally the most abundant genera across most provinces. Notably, *Fusarium* was prominent mainly in Mazandaran, while *Trichoderma* appeared in Qom and Alborz. The presence of endemic genera unique to each province, such as *Microsphaeropsis* in Alborz and *Neofusicoccum* in Mazandaran, suggests that regional climatic and environmental factors strongly influence the composition of endophytic fungal communities associated with *Eucalyptus*. Analyses based on relative abundance (Figs 20 and 21) indicate that Qom province exhibits the highest frequency and diversity of fungal genera, followed by Mazandaran, Tehran, Isfahan, and Alborz, respectively. This pattern highlights the greater richness of endophytic fungi in Qom’s environment compared to the other provinces studied. Specifically, genera like *Alternaria* and *Aspergillus* dominate fungal communities in most provinces, while *Fusarium* shows significant abundance in Mazandaran. These findings emphasize how fungal community structure varies both quantitatively and qualitatively across different regions.

In addition, PCA analysis was conducted to analyze the diversity patterns of fungal genera and visualize their distribution across the studied provinces. The PCA results reveal that the first principal component (PC1) explains 61.24% of the total variance, while the second principal component (PC2) accounts for 38.76%. The analysis shows that among the dominant genera across all five provinces, *Alternaria* is notably abundant, especially in Alborz, where it plays a significant role in shaping the fungal community structure. Furthermore, of the 36 fungal genera identified in this study, 15 genera were found exclusively in Qom province, underscoring the unique and distinct fungal diversity in this region compared to others. These findings highlight clear regional differences in endophytic fungal community composition, influenced likely by local environmental and climatic factors.

## Discussion

In this study, we tested a key idea that the large-scale climate of a region is one of the most important factors determining endophytic fungal diversity. Our results from *Eucalyptus* trees across five provinces of Iran (Alborz, Isfahan, Mazandaran, Tehran, and Qom) strongly support this. Based on the community-level diversity indices such as Shannon-Wiener, Simpson’s dominance, and evenness (Camargo, Margalef, etc.), we found a very clear pattern: fungal diversity was highest in the wet, humid province of Mazandaran (which exhibited the highest fungal richness and evenness) and lowest in the dry provinces of Alborz and Qom. This shows a direct link between climate and diversity. The damp, mild weather in Mazandaran seems to help fungal spores spread, survive, and successfully grow inside the plants, leading to many different species living together. This matches findings from other parts of the world, like a study in tropical forests [67] that also found more endophytes in wetter areas. Our work shows this same rule applies to dry regions, proving that climate is a universal force in shaping the pool of fungi available to plants. Accordingly, Alborz province, characterized by a cooler and drier environment associated with higher elevation, exhibited lower fungal diversity and dominance by a single genus in some tissues. This regional heterogeneity corresponds with broader biogeographical studies indicating that endophyte assemblages are structured by climate, vegetation, elevation gradients, and land use practices [68,69].

A strong climatic signal is evident in the data; however, it is recognized that observed community patterns can, in principle, be influenced by other factors such as stand history, host genetics, or local pollution [9]. However, in the context of our study system, several of these confounding variables are minimized. The sampled *E. camaldulensis* plantations are managed as noncommercial stands without the application of agrochemicals, ruling out a direct chemical treatment effect. Furthermore, while we cannot rule out subtle genetic variation, the use of a single host species across sites reduces the potential for major host genetic differences in endophyte assembly. Therefore, while unmeasured variables like local air quality or fine-scale stand age may contribute to residual variation, the pronounced macroclimatic gradient—spanning arid to humid regions—emerges as the most parsimonious and predominant explanatory variable for the large-scale diversity and compositional patterns we observed.

Looking deeper, we found that climate doesn’t just affect the number of species, but also the types of fungi. In the dry provinces, the fungal communities were simpler and dominated by tough, versatile genera like *Aspergillus* and *Alternaria*. These are like “survivalists” that can handle stress and live in many different environments [69]. This is similar to what were found in *E. globulus* leaves and branches in Uruguay [18] and *E. urophylla* × *E. grandis* hybrids in Brazil [21]. In contrast, the humid climate of Mazandaran supported a more balanced mix of fungi, including specialists like *Neofusicoccum* that are commonly associated with *Eucalyptus* elsewhere such as Australian *Eucalyptus* forests, across leaves, branches, and fruits [70]. This shift from simple communities of generalists in dry areas to complex communities with specialists in wet areas is a major ecological insight from our work.

Elevation gradients are known to influence fungal endophytic community composition by affecting temperature, humidity, and vegetation structure. In this study, Alborz province, located at higher altitude with semi-arid climate, showed lower fungal diversity and dominance by fewer genera. This finding aligns with ecological evidence indicating that increasing elevation imposes abiotic stress and limits colonization by some fungi, thereby reducing richness and altering community assembly [7,67]. In contrast, Tehran and Mazandaran provinces with lower elevation exhibited higher diversity indices and fungal richness. These patterns illuminate the interplay between geographic, climatic, and topographic factors that shape fungal communities and should be considered in future research and conservation efforts.

While climate was the most important factor, the part of the plant we sampled (leaf, branch, or fruit) also mattered—and it mattered in the same way everywhere. Mazandaran yielded the highest number of isolates (358), comprising 150 from leaves, 79 from branches, and 129 from fruits. Tehran contributed 147 isolates (90 leaf, 32 branch, 25 fruit), Qom 103 isolates (62 leaf, 29 branch, 12 fruit), Isfahan 86 isolates (66 leaf, 5 branch, 15 fruit), and Alborz the fewest at 18 isolates (16 leaf, 2 branch, no fruit). This shows that in every single province, leaves had the most diverse fungi, followed by branches, and then fruits. This pattern is logical, as the large surface area and direct aerial exposure of leaves facilitate fungal colonization. In contrast, branches and fruits present more challenging substrates; their tougher structural barriers and potentially higher concentrations of host defensive chemicals [6,17] likely limit fungal establishment, a trend corroborated by other studies on *Eucalyptus* [20]. The novel finding of this study is the remarkable consistency of the colonization hierarchy (leaf > branch > fruit), which persisted regardless of whether the regional fungal species pool was large (as in Mazandaran) or small (as in Alborz). This indicates that host tissue type functions as a strong and reliable secondary filter, consistently structuring endophytic communities across disparate climatic conditions.

It is important to acknowledge that this study was limited to above-ground tissues (leaves, branches, and fruits) and did not include root samples. Root endophytes constitute a critical component of the plant microbiome, playing vital roles in nutrient acquisition, drought tolerance, and defense against soil-borne pathogens, thereby significantly influencing overall plant health [71]. Their communities are shaped by distinct environmental filters, primarily soil properties and rhizosphere dynamics, and often differ substantially in composition and function from those inhabiting aerial tissues. While our findings elucidate consistent, tissue-specific assembly rules for the canopy mycobiome across climates, they represent a partial portrait of the tree’s total fungal assemblage. Future investigations incorporating root samples are therefore needed to achieve a holistic understanding of the complete endophytic consortium in *E. camaldulensis* and its integrated role in tree physiology and adaptation.

Based on the results, leaf and branch tissues of Alborz and Tehran provinces were dominated by *Alternaria* and *Aspergillus*, consistent with reports from *E. urophylla* × *E. grandis* hybrids in Brazil where *Alternaria* predominated leaves and young branches, suggesting preference for photosynthetically active tissue colonization [21]. The abundance of *Penicillium* in Mazandaran leaf and fruit tissues corresponds with Brazilian studies where *Penicillium* was predominantly isolated from leaves and reproductive tissues, indicating a potential protective role in reproductive organs [22]. The genus *Chaetomium*, significant in Qom leaf and fruit tissues, has been reported as a common endophyte in *E. globulus* leaves and twigs in Australia, highlighting its ecological importance across geographically diverse ecosystems [19]. In contrast, *Fusarium* species were isolated at low abundance here compared to reports from China where *Fusarium* was predominantly detected in roots and leaves, likely reflecting geographic and climatic influences on fungal community composition [17]. The genus *Microsphaeropsis* was detected primarily in Alborz branches, which is consistent with reports identifying this genus as a common *E. bark* and woody tissue endophyte in South Africa, indicating tissue specialization [23]. *Trichoderma* was predominantly isolated from branches in Qom and Alborz provinces and is globally recognized as a biocontrol agent strongly associated with woody tissues of various *Eucalyptus* species [72,73]. Fruit tissues exhibited lower diversity and were frequently dominated by a few species such as *Alternaria* and *Chaetomium*. Similar patterns have been reported in *E. grandis*, where fruit endophytes were less diverse and dominated by fewer taxa than leaves, likely due to selective colonization linked to tissue chemical defenses [20]. The minimal fruit endophyte diversity in Alborz corresponds with prior reports indicating that fruit tissues generally limit fungal colonization compared to leaves and branches in *Eucalyptus* species worldwide [6].

We also noted interesting patterns in where specific fungi were found. Genera like *Cladosporium* and *Penicillium* were found in all sampling sites, showing they are widespread generalists. This fits with their reported presence in *Eucalyptus* studies from South America to Australia [19,21]. On the other hand, a fungus like *Neofusicoccum* was very common in Mazandaran but absent from the dry provinces, matching its known preference for wetter environments globally [70]. Finding Mucoromycota only in Isfahan shows that some rare fungi have very specific locations. The presence of useful genera like *Trichoderma* (known for biocontrol) in certain areas also highlights the potential to find fungi with special properties that are adapted to local conditions.

The community patterns observed in *Eucalyptus* are not exceptional but reflect broader ecological principles. Studies across diverse plant systems, including Iranian native flora, report similar drivers. For example, research on Iranian *Ferula* [74] and *Salvia* [75] species identified climate and geography as key determinants of endophyte composition. The findings for *E. camaldulensis* align with those of Safaie et al. [74], particularly regarding the dominance of the phylum Ascomycota and tissue-specific colonization patterns, where leaves (in *Eucalyptus*) and roots (in *Ferula*) hosted richer fungal diversity, underscoring the influence of host anatomy. Furthermore, both studies demonstrate that geographic and climatic factors significantly structure communities, with humid regions supporting higher richness and evenness. Jahromi et al. [75] similarly found that humidity, more than temperature, primarily influenced endophyte richness in *Salvia multicaulis*, and reported a community overwhelmingly dominated by Ascomycota (specifically Eurotiomycetes, Sordariomycetes, and Dothideomycetes), with minor contributions from Mucoromycota. Globally, comparable climatic drivers are evident. Oita et al. [67] found tropical endophyte richness increased with precipitation and was structured by temperature seasonality, showing high local endemism across climate gradients. Zimmerman and Vitousek [76] reported that compositional variation in leaf endophytes of *Metrosideros polymorpha* in Hawaii correlated strongly with temperature and rainfall. Furthermore, a study on the invasive plant *Ageratina adenophora* revealed that its cultivable endophytic fungi exhibited geographic and tissue-specific variation, partially overlapping with environmental sources, highlighting dynamic interactions shaped by both plant function and local conditions [77]. This consistent evidence across disparate hosts and ecosystems suggests that the assembly of endophytic fungal communities follows general, predictable rules, with climate acting as the primary and most influential filter.

## Conclusion

In conclusion, this study demonstrates that the assembly of endophytic fungal communities in *E. camaldulensis* follows a clear hierarchy, with macro-climate serving as the primary determinant of regional diversity and composition, and host tissue type acting as a consistent secondary filter. The dramatic decline in fungal richness and the shift from stress-tolerant generalists in arid provinces to diverse specialists in humid one’s underscore climate’s overriding influence. This framework not only provides the first comprehensive baseline for endophytes in Iranian *Eucalyptus* but also carries critical implications: it suggests that increasing aridity from climate change could erode this hidden diversity, potentially impacting tree health. Furthermore, the distinct fungal communities isolated represent a valuable biotechnological resource, highlighting the importance of conserving these cryptic symbiotic systems.

## Supporting information

S1 Table Climate information for each region. (DOCX)

S2 Table List of fungal isolates identified in this study, including genus and species, tissue source, province of collection, and accession numbers for ITS and *tef-1α* gene sequences submitted to GenBank. (DOCX)
