# Supplementary material for: Biodiversity of Eucalyptus endophytic fungi across different climates in Iran
Source: PLoS One. 2026 Jul 23;21(7):e0345700. doi: 10.1371/journal.pone.0345700 (PMC13395448; doi:10.1371/journal.pone.0345700)
Supplement: S1 Table — (DOCX) [file pone.0345700.s001.docx]

**S1 Table. Climate information for each region.**

| **Province** | **Climate Type** | **Avg. Temp (°C)** | **Precipitation (mm)** | **Elevation (m)** |
| --- | --- | --- | --- | --- |
| Alborz | Semi-arid to temperate | 14.4 | Moderate | 1750 |
| Isfahan | Semi-arid continental | 17 | Low | 1607 |
| Mazandaran | Humid subtropical | 20 | High | 863 |
| Qom | Xeric, Desertic | 21 | Low | 943 |
| Tehran | Semi-arid | 17.4 | Moderate | 1539 |
