# Supplementary material for: Biodiversity of Eucalyptus endophytic fungi across different climates in Iran
Source: PLoS One. 2026 Jul 23;21(7):e0345700. doi: 10.1371/journal.pone.0345700 (PMC13395448; doi:10.1371/journal.pone.0345700)
Supplement: S2 Table — (DOCX) [file pone.0345700.s002.docx]

**S2 Table. List of fungal isolates identified in this study, including genus and species, tissue source, province of collection, and accession numbers for ITS and *tef-1α* gene sequences submitted to GenBank.**

| Isolates | Genus and species | Tissue | Province | Accession | GenBank |
| --- | --- | --- | --- | --- | --- |
|  |  |  |  |  | **ITS** |
| BL4 | *Valsonectria* sp*.* | Leaf | Tehran | ABRIICC 10423 | PQ496771 |
| DL2 | *Alternaria* sp. 1 | Leaf | Tehran | ABRIICC 10425 | PQ496772 |
| VL6 | *Alternaria* sp. 2 | Leaf | Mazandaran | ABRIICC 10443 | PQ496773 |
| XF1 | *Alternaria* sp. 3 | Fruit | Mazandaran | ABRIICC 10444 | PQ496774 |
| JL2 | *Alternaria* sp. 4 | Leaf | Isfahan | ABRIICC 10430 | PQ496775 |
| RF2 | *Alternaria* sp. 5 | Fruit | Mazandaran | ABRIICC 10438 | PQ496776 |
| NS1 | *Aspergillus* sp. 1 | Branch | Mazandaran | ABRIICC 10435 | PQ496777 |
| 18L1 | *Aspergillus* sp. 2 | Leaf | Qom | ABRIICC 10421 | PQ496778 |
| 15F2 | *Aspergillus* sp. 3 | Fruit | Tehran | ABRIICC 10419 | PQ496779 |
| VL1A | *Aspergillus* sp. 4 | Leaf | Mazandaran | ABRIICC 10441 | PQ496780 |
| 9L1 | *Aspergillus* sp. 5 | Leaf | Alborz | ABRIICC 10415 | PQ496781 |
| YL1 | *Bipolaris* sp. 1 | Leaf | Tehran | ABRIICC 10446 | PQ496782 |
| OL2 | *Bipolaris* sp. 2 | Leaf | Tehran | ABRIICC 10436 | PQ496783 |
| FL1 | *Cladosporium* sp. 1 | Leaf | Qom | ABRIICC 10427 | PQ496784 |
| SS2 | *Cladosporium* sp. 2 | Branch | Mazandaran | ABRIICC 10440 | PQ496785 |
| 15F3 | *Cytospora* sp. | Branch | Tehran | ABRIICC 10420 | PQ496786 |
| LS1 | *Didymella* sp. | Branch | Mazandaran | ABRIICC 10433 | PQ496787 |
| JL3 | *Didymosphaeria* sp. | Leaf | Isfahan | ABRIICC 10431 | PQ496788 |
| KF1 | *Didymosphaeria* sp. | Fruit | Qom | ABRIICC 10432 | PQ496789 |
| XS1 | *Didymosphaeria* sp. | Branch | Mazandaran | ABRIICC 10445 | PQ496790 |
| HL2 | *Gymnoascus* sp. | Fruit | Isfahan | ABRIICC 10428 | PQ496791 |
| 13F2 | *Iodophanus* sp. | Fruit | Tehran | ABRIICC 10416 | PQ496792 |
| 13F3 | *Iodophanus* sp. | Branch | Tehran | ABRIICC 10447 | PQ496793 |
| 14S2 | *Microsphaeropsis* sp. | Branch | Tehran | ABRIICC 10418 | PQ496794 |
| MS1 | *Neofusicoccum* sp. | Branch | Mazandaran | ABRIICC 10434 | PQ496795 |
| SL1 | *Neofusicoccum* sp. | Leaf | Mazandaran | - | PQ496796 |
| JF1 | *Schizothecium* sp. | Fruit | Isfahan | - | PQ496797 |
| EL1 | *Niesslia* sp. | Leaf | Qom | - | PQ496798 |
| VL3 | *Penicillium* sp. 1 | Leaf | Mazandaran | ABRIICC 10442 | PQ496799 |
| PS1 | *Penicillium* sp. 2 | Branch | Tehran | ABRIICC 10437 | PQ496800 |
| 13S1 | *Penicillium* sp. 3 | Branch | Tehran | ABRIICC 10417 | PQ496801 |
| CS1 | *Peziza* sp. | Branch | Tehran | ABRIICC 10424 | PQ496802 |
| BL3 | *Ulocladium* sp. | Leaf | Tehran | ABRIICC 10422 | PQ496803 |
| DL4 | *Chaetomium globosum* | Leaf | Tehran | ABRIICC 10383 | PP320329 |
| VL3 | *Pseudosydowia eucalypti* | Leaf | Mazandaran | ABRIICC 10385 | PP320331 |
| XL4 | *Phaeophleospora eucalypticola* | Leaf | Mazandaran | ABRIICC 10384 | PP320330 |
|  |  |  |  |  | ***tef-1α*** |
| 8S1 | *Trichoderma longibrachiatum* | Branch | Alborz | ABRIICC 10382 | PP314025 |
| KL1 | *Trichoderma longibrachiatum* | Leaf | Mazandaran | ABRIICC 10381 | PP314024 |
