# Supplementary material for: Biodiversity of Eucalyptus endophytic fungi across different climates in Iran
Source: PLoS One. 2026 Jul 23;21(7):e0345700. doi: 10.1371/journal.pone.0345700 (PMC13395448; doi:10.1371/journal.pone.0345700)
Supplement: Attachment — Submitted filename: Decsion for Revision - Mjaor Revision.docx [file pone.0345700.s003.docx]

**Manuscript Number PONE-D-25-55331** *“ Biodiversity of Eucalyptus endophytic fungi across different climates in Iran”*

**Dear Dr. Ebrahimi**

Thank you for submitting your manuscript to PLOS ONE. After careful consideration, we feel that it has merit but does not fully meet PLOS ONE’s publication criteria as it currently stands. Therefore, we invite you to submit a revised version of the manuscript that addresses the points raised during the review process.

We have received the reports from our advisors on your manuscript**, “***Biodiversity of Eucalyptus endophytic fungi across different climates in Iran***” (PONE-D-25-55331)”**submitted to PLOS One.

Based on the advice received, Your Manuscript can be considered for further evaluation after you will accept & carried out the corrections as suggested by the reviewer in your Re submission (s).

Below, please find the reviewers' comments for your perusal. You are kindly requested to also check the website for possible reviewer attachment (s).

While submitting, please check the filled in author data carefully and update them if applicable - they need to be complete and correct in order for the revision to be processed further.

Please note: When uploading your revised manuscript files, please make sure only to submit your editable source files (i.e. Word, Tex).

Please submit your revised manuscript online by using the Editorial Manager system which can be accessed at:

I am looking forward to receiving your revised manuscript before 17^th^ December, 2025.
In order to add the due date to your electronic calendar, please open the attached file.

**With kind regards,**
Academic Editor
PLOS One

**Reviewers Comments:**

**Reviewer 1.**

The authors examined the diversity of endophytic fungi in Eucalyptus across five provinces in Iran, representing a range of climatic conditions. They isolated over 700 fungal strains from various tissues of Eucalyptus. Their analysis indicates that climatic conditions are the primary factor shaping fungal diversity. This is an extensive sampling effort. A key limitation is that it remains unclear whether the isolated fungi are true endophytes, as colonization assays were not performed to confirm their endophytic status. I noticed that some sections of the manuscript use an informal tone. To maintain a professional, academic style, I recommend revising these sentences using formal language. The paper is technically sound and makes a valuable contribution to the existing literature on fungal endophytes on woody plants.

**Major comments:**

Introduction: The authors mention that fungal communities in Eucalyptus have been studied various parts of the world but not in Iran. This statement needs additional context and supporting references. I would suggest adding a brief summary of key findings from previous studies on fungal endophytes in Eucalyptus and clarify why examining these communities in Iran is important.

Discussion: The study focuses on fungal communities in leaves, fruits, stems, and branches; however, roots were not included. Root associated endophytic fungi play important roles in overall plant health and their communities may differ substantially from those in above-ground plant parts. Although it may not be feasible to add root samples at this stage, the authors should acknowledge this limitation and briefly discuss the ecological importance of root endophytes.

Lines 77 -78 and Lines 218-219: The control group used in this study is WA plates without plant samples. No fungal growth was observed on the control plates, indicating that the medium and handling conditions were sterile. This does not confirm the effectiveness of the surface-sterilization procedure, which would require plating the final rinse of water after surface sterilization or use tissue imprints.

**Minor comments:**

Line 428, Line 500, Line 535: I have noticed sentences with informal tone in many sections of the manuscript. I would suggest revising the manuscript for a more formal and academic tone.

Line 528: “Genera like Cladosporium and Penicillium were found everywhere…”. Please be more specific about what ‘everywhere’ refers to (eg. all sampling sites, all plant tissues, or all climates).

Including a map of Iran showing the sampling locations as a supplementary figure would be helpful for the readers.

**Reviewer 2 :**

Biodiversity of Eucalyptus endophytic fungi across different climates in Iran

The present study isolated 712 fungal species from E. camaldulensis across five provinces with climates that range from arid to humid in Iran. The humid Mazandaran province exhibited the highest diversity and colonization frequency, while arid Alborz and Qom provinces showed lower diversity dominated by genera such as *Alternaria and Aspergillus*. On the other hand, the humid site had more balanced communities, including Neofusicoccum and Didymosphaeria. Leaves were the primary niche for diverse fungal communities, followed by branches and fruits. Beta-diversity analyses indicated regional and tissue-specific turnover influenced by climate and host tissue. This work establishes a baseline for E. camaldulensis endophytes in Iran and highlights those genera which may have potential for biocontrol and biotechnological use under climate change.

The article describes a cultivation-based survey demonstrating that macro-climate is the main factor influencing Eucalyptus endophytic fungal diversity in Iran, with host tissue type as a secondary filter. However, it notes several framing, methodological clarity, and interpretation issues that need to be improved for publication rigor.

The manuscript may be accepted after the following revisions.
Include in a brief table the climatic and geographic parameters of each province, such as mean annual precipitation -MAP , mean annual precipitation-MAT, humidity, and elevation, preferably from the same period of time as the sampling.
Employ these variables to better underpin the arid–humid gradient and place the interpretation of "macro-climate" into a more quantitative context than simple descriptive terms.
In the Discussion, make sure to differentiate those factors that could be reasonably attributed to climate from those that may also reflect stand history, host genetic diversity, air quality, or the deployment of agrochemicals; currently, climate is sometimes posed as just about the only driver.
Improvise the figures with clarity of visibility
All isolates of WA → PDA have only culturable isolates, which automatically results in underrepresentation of most endophyte species.
It should be noted whether any attempt has been made at modifying conditions of media or incubation in efforts to improve culturable diversity.
The number of samples in provinces is imbalanced (for example, Tehran has 17 samples of trees in contrast to 4 in Alborz and 7 in Qom).
There can be an assessment of diversity within the tissues of all provinces and then an analysis of the importance of their distribution.

**Reviewer 3 :**

The study represents the diversity of endophytic fungi in Eucalyptus under various climatic conditions. The work is extensive and well-presented both technically and scientifically.
Have you submitted the cultures in culture collection?
Have you submitted the ITS sequences in GenBank?
