# Supplementary material for: Biodiversity of Eucalyptus endophytic fungi across different climates in Iran
Source: PLoS One. 2026 Jul 23;21(7):e0345700. doi: 10.1371/journal.pone.0345700 (PMC13395448; doi:10.1371/journal.pone.0345700)
Supplement: Attachment — Submitted filename: reviewers response.docx [file pone.0345700.s005.docx]

Dear editor,

we are grateful for the very valuable reviewer comments. We have now thoroughly revised the manuscript addressing the comments.

With best regards,

Leila Ebrahimi, Ph.D.

**Reviewers Comments:**

**Reviewer 1.**
**Major comments:**

Introduction: The authors mention that fungal communities in Eucalyptus have been studied various parts of the world but not in Iran. This statement needs additional context and supporting references. I would suggest adding a brief summary of key findings from previous studies on fungal endophytes in Eucalyptus and clarify why examining these communities in Iran is important.

We have incorporated relevant findings from previous studies and expanded the introduction part on the importance of investigating *Eucalyptus* endophytes.

Discussion: The study focuses on fungal communities in leaves, fruits, stems, and branches; however, roots were not included. Root associated endophytic fungi play important roles in overall plant health and their communities may differ substantially from those in above-ground plant parts. Although it may not be feasible to add root samples at this stage, the authors should acknowledge this limitation and briefly discuss the ecological importance of root endophytes.

We have added both in discussion.

Lines 77 -78 and Lines 218-219: The control group used in this study is WA plates without plant samples. No fungal growth was observed on the control plates, indicating that the medium and handling conditions were sterile. This does not confirm the effectiveness of the surface-sterilization procedure, which would require plating the final rinse of water after surface sterilization or use tissue imprints.

Thank you for this observation. We have corrected it accordingly.

**Minor comments:**

Line 428, Line 500, Line 535: I have noticed sentences with informal tone in many sections of the manuscript. I would suggest revising the manuscript for a more formal and academic tone.

Corrected.

Line 528: “Genera like Cladosporium and Penicillium were found everywhere…”. Please be more specific about what ‘everywhere’ refers to (eg. all sampling sites, all plant tissues, or all climates).

Corrected.

Including a map of Iran showing the sampling locations as a supplementary figure would be helpful for the readers.

We have added a map showing the sampling locations as S1 Figure.

**Reviewer 2:**

Include in a brief table the climatic and geographic parameters of each province, such as mean annual precipitation -MAP, mean annual precipitation-MAT, humidity, and elevation, preferably from the same period of time as the sampling. Employ these variables to better underpin the arid–humid gradient and place the interpretation of "macro-climate" into a more quantitative context than simple descriptive terms.

We have added a supplementary table (S1 Table) with information for each province.

In the Discussion, make sure to differentiate those factors that could be reasonably attributed to climate from those that may also reflect stand history, host genetic diversity, air quality, or the deployment of agrochemicals; currently, climate is sometimes posed as just about the only driver.

Thank you for this important comment, which encourages greater precision in attributing ecological drivers. We have revised the Discussion to acknowledge that factors such as stand history, host genetics, and local conditions can, in theory, influence endophyte communities.

Improvise the figures with clarity of visibility.

Thanks. We improved the quality of the figures.

All isolates of WA → PDA have only culturable isolates, which automatically results in underrepresentation of most endophyte species. It should be noted whether any attempt has been made at modifying conditions of media or incubation in efforts to improve culturable diversity.

This is an excellent idea; however, we have not implemented it.

The number of samples in provinces is imbalanced (for example, Tehran has 17 samples of trees in contrast to 4 in Alborz and 7 in Qom). There can be an assessment of diversity within the tissues of all provinces and then an analysis of the importance of their distribution.

Thank you for raising this valid point regarding the uneven sampling across provinces. We acknowledge the imbalance, which resulted from the natural, patchy distribution of *Eucalyptus* plantations across Iran's varied climates. We chose to sample all available, healthy stands within our target climatic gradient, prioritizing ecological representativeness over numerical parity.

We wish to assure you that the diversity metrics central to our conclusions are robust to this sampling heterogeneity for the following reasons:

Use of sample-size corrected indices: our analysis relies on diversity indices explicitly designed to be comparable across different sampling efforts. The Shannon-Wiener (H') and Simpson's (1-D) indices incorporate species richness and relative abundance, while Pielou's Evenness (J') measures the distribution of individuals. Crucially, the Chao1 estimator was used to estimate total species richness, a non-parametric metric that is reliable for smaller samples as it is based on the frequency of rare species.

Focus on proportional colonization and relative patterns: our key findings are based on proportional metrics (Colonization Frequency, Relative Abundance) and consistent patterns (e.g., the universal leaf > branch > fruit hierarchy; the climate-driven richness gradient from humid to arid provinces). These patterns are evident in the standardized data and are not an artifact of varying tree counts.

Consistency with ecological expectation: the observed pattern—highest diversity in humid Mazandaran and lowest in arid Alborz/Qom—aligns strongly with established global principles of microbial biogeography. The fact that this clear gradient emerges despite sampling constraints reinforces, rather than weakens, the primary role of climate as a driver.

**Reviewer 3:**

The study represents the diversity of endophytic fungi in Eucalyptus under various climatic conditions. The work is extensive and well-presented both technically and scientifically.
Have you submitted the cultures in culture collection? Have you submitted the ITS sequences in GenBank?

Yes, we have deposited the cultures in a collection and the corresponding sequences in NCBI, with all related accession numbers and strain data provided in S2 Table.

Thank you so much again for your invaluable comments.

Best Regards.
