# Supplementary material for: Biodiversity of Eucalyptus endophytic fungi across different climates in Iran
Source: PLoS One. 2026 Jul 23;21(7):e0345700. doi: 10.1371/journal.pone.0345700 (PMC13395448; doi:10.1371/journal.pone.0345700)
Supplement: Peer review history

# Peer review: Decision Letter 0

**DOI:** 10.1371/journal.pone.0345700.r001

**Authors:** Phalisteen Sultan

13 Jan 2026

PONE-D-25-55331

Biodiversity of Eucalyptus endophytic fungi across different climates in Iran

PLOS One

Dear Dr. Ebrahimi,

Thank you for submitting your manuscript to PLOS ONE. After careful consideration, we feel that it has merit but does not fully meet PLOS ONE’s publication criteria as it currently stands. Therefore, we invite you to submit a revised version of the manuscript that addresses the points raised during the review process.

We have received the reports from our advisors on your manuscript, “Biodiversity of Eucalyptus endophytic fungi across different climates in Iran” (PONE-D-25-55331)”submitted to PLOS One.

Based on the advice received, Your Manuscript can be considered for further evaluation after you will accept & carried out the corrections as suggested by the reviewer in your Re submission (s).

Below, please find the reviewers' comments for your perusal. You are kindly requested to also check the website for possible reviewer attachment (s).

While submitting, please check the filled in author data carefully and update them if applicable - they need to be complete and correct in order for the revision to be processed further.

Please note: When uploading your revised manuscript files, please make sure only to submit your editable source files (i.e. Word, Tex).

Please submit your revised manuscript by Feb 27 2026 11:59PM. If you will need more time than this to complete your revisions, please reply to this message or contact the journal office at plosone@plos.org. When you're ready to submit your revision, log on to https://www.editorialmanager.com/pone/ and select the 'Submissions Needing Revision' folder to locate your manuscript file.

Please include the following items when submitting your revised manuscript:

- A letter that responds to each point raised by the academic editor and reviewer(s). You should upload this letter as a separate file labeled 'Response to Reviewers'.
- A marked-up copy of your manuscript that highlights changes made to the original version. You should upload this as a separate file labeled 'Revised Manuscript with Track Changes'.
- An unmarked version of your revised paper without tracked changes. You should upload this as a separate file labeled 'Manuscript'.

If you would like to make changes to your financial disclosure, please include your updated statement in your cover letter. Guidelines for resubmitting your figure files are available below the reviewer comments at the end of this letter.

If applicable, we recommend that you deposit your laboratory protocols in protocols.io to enhance the reproducibility of your results. Protocols.io assigns your protocol its own identifier (DOI) so that it can be cited independently in the future. For instructions see: https://journals.plos.org/plosone/s/submission-guidelines#loc-laboratory-protocols. Additionally, PLOS ONE offers an option for publishing peer-reviewed Lab Protocol articles, which describe protocols hosted on protocols.io. Read more information on sharing protocols at https://plos.org/protocols?utm_medium=editorial-email&utm_source=authorletters&utm_campaign=protocols.

We look forward to receiving your revised manuscript.

Kind regards,

Phalisteen Sultan

Academic Editor

PLOS One

Journal Requirements:

When submitting your revision, we need you to address these additional requirements.

1. Please ensure that your manuscript meets PLOS ONE's style requirements, including those for file naming. The PLOS ONE style templates can be found at

https://journals.plos.org/plosone/s/file?id=wjVg/PLOSOne_formatting_sample_main_body.pdf and

https://journals.plos.org/plosone/s/file?id=ba62/PLOSOne_formatting_sample_title_authors_affiliations.pdf

2. In your Methods section, please provide additional information regarding the permits you obtained for the work. Please ensure you have included the full name of the authority that approved the field site access and, if no permits were required, a brief statement explaining why.

3. When completing the data availability statement of the submission form, you indicated that you will make your data available on acceptance. We strongly recommend all authors decide on a data sharing plan before acceptance, as the process can be lengthy and hold up publication timelines. Please note that, though access restrictions are acceptable now, your entire data will need to be made freely accessible if your manuscript is accepted for publication. This policy applies to all data except where public deposition would breach compliance with the protocol approved by your research ethics board. If you are unable to adhere to our open data policy, please kindly revise your statement to explain your reasoning and we will seek the editor's input on an exemption. Please be assured that, once you have provided your new statement, the assessment of your exemption will not hold up the peer review process.

4. Thank you for stating the following financial disclosure:

The author(s) received no specific funding for this work.

At this time, please address the following queries:

a) Please clarify the sources of funding (financial or material support) for your study. List the grants or organizations that supported your study, including funding received from your institution.

b) State what role the funders took in the study. If the funders had no role in your study, please state: “The funders had no role in study design, data collection and analysis, decision to publish, or preparation of the manuscript.”

c) If any authors received a salary from any of your funders, please state which authors and which funders.

d) If you did not receive any funding for this study, please state: “The authors received no specific funding for this work.”

Please include your amended statements within your cover letter; we will change the online submission form on your behalf.

5. Thank you for stating the following in the Acknowledgments Section of your manuscript:

We gratefully acknowledge the Iran National Science Foundation (INSF), and University of Tehran, Iran, for financial support.

We note that you have provided funding information that is not currently declared in your Funding Statement. However, funding information should not appear in the Acknowledgments section or other areas of your manuscript. We will only publish funding information present in the Funding Statement section of the online submission form.

Please remove any funding-related text from the manuscript and let us know how you would like to update your Funding Statement. Currently, your Funding Statement reads as follows:

The author(s) received no specific funding for this work.

Please include your amended statements within your cover letter; we will change the online submission form on your behalf.

6. Please include captions for your Supporting Information files at the end of your manuscript, and update any in-text citations to match accordingly. Please see our Supporting Information guidelines for more information: http://journals.plos.org/plosone/s/supporting-information.

7. If the reviewer comments include a recommendation to cite specific previously published works, please review and evaluate these publications to determine whether they are relevant and should be cited. There is no requirement to cite these works unless the editor has indicated otherwise.

Additional Editor Comments:

Manuscript Number PONE-D-25-55331 “ Biodiversity of Eucalyptus endophytic fungi across different climates in Iran”

Dear Dr. Ebrahimi

Thank you for submitting your manuscript to PLOS ONE. After careful consideration, we feel that it has merit but does not fully meet PLOS ONE’s publication criteria as it currently stands. Therefore, we invite you to submit a revised version of the manuscript that addresses the points raised during the review process.

We have received the reports from our advisors on your manuscript, “Biodiversity of Eucalyptus endophytic fungi across different climates in Iran” (PONE-D-25-55331)”submitted to PLOS One.

Based on the advice received, Your Manuscript can be considered for further evaluation after you will accept & carried out the corrections as suggested by the reviewer in your Re submission (s).

Below, please find the reviewers' comments for your perusal. You are kindly requested to also check the website for possible reviewer attachment (s).

While submitting, please check the filled in author data carefully and update them if applicable - they need to be complete and correct in order for the revision to be processed further.

Please note: When uploading your revised manuscript files, please make sure only to submit your editable source files (i.e. Word, Tex).

With kind regards,

Phalisteen Sultan, Ph.D.

Academic Editor

PLOS One

Reviewers Comments:

Reviewer 1.

The authors examined the diversity of endophytic fungi in Eucalyptus across five provinces in Iran, representing a range of climatic conditions. They isolated over 700 fungal strains from various tissues of Eucalyptus. Their analysis indicates that climatic conditions are the primary factor shaping fungal diversity. This is an extensive sampling effort. A key limitation is that it remains unclear whether the isolated fungi are true endophytes, as colonization assays were not performed to confirm their endophytic status. I noticed that some sections of the manuscript use an informal tone. To maintain a professional, academic style, I recommend revising these sentences using formal language. The paper is technically sound and makes a valuable contribution to the existing literature on fungal endophytes on woody plants.

Major comments:

Introduction: The authors mention that fungal communities in Eucalyptus have been studied various parts of the world but not in Iran. This statement needs additional context and supporting references. I would suggest adding a brief summary of key findings from previous studies on fungal endophytes in Eucalyptus and clarify why examining these communities in Iran is important.

Discussion: The study focuses on fungal communities in leaves, fruits, stems, and branches; however, roots were not included. Root associated endophytic fungi play important roles in overall plant health and their communities may differ substantially from those in above-ground plant parts. Although it may not be feasible to add root samples at this stage, the authors should acknowledge this limitation and briefly discuss the ecological importance of root endophytes.

Lines 77 -78 and Lines 218-219: The control group used in this study is WA plates without plant samples. No fungal growth was observed on the control plates, indicating that the medium and handling conditions were sterile. This does not confirm the effectiveness of the surface-sterilization procedure, which would require plating the final rinse of water after surface sterilization or use tissue imprints.

Minor comments:

Line 428, Line 500, Line 535: I have noticed sentences with informal tone in many sections of the manuscript. I would suggest revising the manuscript for a more formal and academic tone.

Line 528: “Genera like Cladosporium and Penicillium were found everywhere…”. Please be more specific about what ‘everywhere’ refers to (eg. all sampling sites, all plant tissues, or all climates).

Including a map of Iran showing the sampling locations as a supplementary figure would be helpful for the readers.

Reviewer 2 :

Biodiversity of Eucalyptus endophytic fungi across different climates in Iran

The present study isolated 712 fungal species from E. camaldulensis across five provinces with climates that range from arid to humid in Iran. The humid Mazandaran province exhibited the highest diversity and colonization frequency, while arid Alborz and Qom provinces showed lower diversity dominated by genera such as Alternaria and Aspergillus. On the other hand, the humid site had more balanced communities, including Neofusicoccum and Didymosphaeria. Leaves were the primary niche for diverse fungal communities, followed by branches and fruits. Beta-diversity analyses indicated regional and tissue-specific turnover influenced by climate and host tissue. This work establishes a baseline for E. camaldulensis endophytes in Iran and highlights those genera which may have potential for biocontrol and biotechnological use under climate change.

The article describes a cultivation-based survey demonstrating that macro-climate is the main factor influencing Eucalyptus endophytic fungal diversity in Iran, with host tissue type as a secondary filter. However, it notes several framing, methodological clarity, and interpretation issues that need to be improved for publication rigor.

The manuscript may be accepted after the following revisions.

Include in a brief table the climatic and geographic parameters of each province, such as mean annual precipitation -MAP , mean annual precipitation-MAT, humidity, and elevation, preferably from the same period of time as the sampling.

Employ these variables to better underpin the arid–humid gradient and place the interpretation of "macro-climate" into a more quantitative context than simple descriptive terms.

In the Discussion, make sure to differentiate those factors that could be reasonably attributed to climate from those that may also reflect stand history, host genetic diversity, air quality, or the deployment of agrochemicals; currently, climate is sometimes posed as just about the only driver.

Improvise the figures with clarity of visibility

All isolates of WA → PDA have only culturable isolates, which automatically results in underrepresentation of most endophyte species.

It should be noted whether any attempt has been made at modifying conditions of media or incubation in efforts to improve culturable diversity.

The number of samples in provinces is imbalanced (for example, Tehran has 17 samples of trees in contrast to 4 in Alborz and 7 in Qom).

There can be an assessment of diversity within the tissues of all provinces and then an analysis of the importance of their distribution.

Reviewer 3 :

The study represents the diversity of endophytic fungi in Eucalyptus under various climatic conditions. The work is extensive and well-presented both technically and scientifically.

Have you submitted the cultures in culture collection?

Have you submitted the ITS sequences in GenBank?

[Note: HTML markup is below. Please do not edit.]

Reviewers' comments:

Reviewer's Responses to Questions

**Comments to the Author**

1. Is the manuscript technically sound, and do the data support the conclusions?

The manuscript must describe a technically sound piece of scientific research with data that supports the conclusions. Experiments must have been conducted rigorously, with appropriate controls, replication, and sample sizes. The conclusions must be drawn appropriately based on the data presented.

Reviewer #1: Yes

Reviewer #2: Yes

Reviewer #3: Yes

**********

2. Has the statistical analysis been performed appropriately and rigorously?

Reviewer #1: Yes

Reviewer #2: Yes

Reviewer #3: Yes

**********

3. Have the authors made all data underlying the findings in their manuscript fully available?

The PLOS Data policy requires authors to make all data underlying the findings described in their manuscript fully available without restriction, with rare exception (please refer to the Data Availability Statement in the manuscript PDF file). The data should be provided as part of the manuscript or its supporting information, or deposited to a public repository. For example, in addition to summary statistics, the data points behind means, medians and variance measures should be available. If there are restrictions on publicly sharing data—e.g. participant privacy or use of data from a third party—those must be specified.

Reviewer #1: Yes

Reviewer #2: Yes

Reviewer #3: Yes

**********

4. Is the manuscript presented in an intelligible fashion and written in standard English?

PLOS ONE does not copyedit accepted manuscripts, so the language in submitted articles must be clear, correct, and unambiguous. Any typographical or grammatical errors should be corrected at revision, so please note any specific errors here.

Reviewer #1: No

Reviewer #2: Yes

Reviewer #3: Yes

**********

5. Review Comments to the Author

Please use the space provided to explain your answers to the questions above. You may also include additional comments for the author, including concerns about dual publication, research ethics, or publication ethics. (Please upload your review as an attachment if it exceeds 20,000 characters)

Reviewer #1: The authors examined the diversity of endophytic fungi in Eucalyptus across five provinces in Iran, representing a range of climatic conditions. They isolated over 700 fungal strains from various tissues of Eucalyptus. Their analysis indicates that climatic conditions are the primary factor shaping fungal diversity. This is an extensive sampling effort. A key limitation is that it remains unclear whether the isolated fungi are true endophytes, as colonization assays were not performed to confirm their endophytic status. I noticed that some sections of the manuscript use an informal tone. To maintain a professional, academic style, I recommend revising these sentences using formal language. The paper is technically sound and makes a valuable contribution to the existing literature on fungal endophytes on woody plants.

Major comments:

Introduction: The authors mention that fungal communities in Eucalyptus have been studied various parts of the world but not in Iran. This statement needs additional context and supporting references. I would suggest adding a brief summary of key findings from previous studies on fungal endophytes in Eucalyptus and clarify why examining these communities in Iran is important.

Discussion: The study focuses on fungal communities in leaves, fruits, stems, and branches; however, roots were not included. Root associated endophytic fungi play important roles in overall plant health and their communities may differ substantially from those in above-ground plant parts. Although it may not be feasible to add root samples at this stage, the authors should acknowledge this limitation and briefly discuss the ecological importance of root endophytes.

Lines 77 -78 and Lines 218-219: The control group used in this study is WA plates without plant samples. No fungal growth was observed on the control plates, indicating that the medium and handling conditions were sterile. This does not confirm the effectiveness of the surface-sterilization procedure, which would require plating the final rinse of water after surface sterilization or use tissue imprints.

Minor comments:

Line 428, Line 500, Line 535: I have noticed sentences with informal tone in many sections of the manuscript. I would suggest revising the manuscript for a more formal and academic tone.

Line 528: “Genera like Cladosporium and Penicillium were found everywhere…”. Please be more specific about what ‘everywhere’ refers to (eg. all sampling sites, all plant tissues, or all climates).

Including a map of Iran showing the sampling locations as a supplementary figure would be helpful for the readers.

Reviewer #2: Biodiversity of Eucalyptus endophytic fungi across different climates in Iran

The present study isolated 712 fungal species from E. camaldulensis across five provinces with climates that range from arid to humid in Iran. The humid Mazandaran province exhibited the highest diversity and colonization frequency, while arid Alborz and Qom provinces showed lower diversity dominated by genera such as Alternaria and Aspergillus. On the other hand, the humid site had more balanced communities, including Neofusicoccum and Didymosphaeria. Leaves were the primary niche for diverse fungal communities, followed by branches and fruits. Beta-diversity analyses indicated regional and tissue-specific turnover influenced by climate and host tissue. This work establishes a baseline for E. camaldulensis endophytes in Iran and highlights those genera which may have potential for biocontrol and biotechnological use under climate change.

The article describes a cultivation-based survey demonstrating that macro-climate is the main factor influencing Eucalyptus endophytic fungal diversity in Iran, with host tissue type as a secondary filter. However, it notes several framing, methodological clarity, and interpretation issues that need to be improved for publication rigor.

The manuscript may be accepted after the following revisions.

Include in a brief table the climatic and geographic parameters of each province, such as mean annual precipitation -MAP , mean annual precipitation-MAT, humidity, and elevation, preferably from the same period of time as the sampling.

Employ these variables to better underpin the arid–humid gradient and place the interpretation of "macro-climate" into a more quantitative context than simple descriptive terms.

In the Discussion, make sure to differentiate those factors that could be reasonably attributed to climate from those that may also reflect stand history, host genetic diversity, air quality, or the deployment of agrochemicals; currently, climate is sometimes posed as just about the only driver.

Improvise the figures with clarity of visibility

All isolates of WA → PDA have only culturable isolates, which automatically results in underrepresentation of most endophyte species.

It should be noted whether any attempt has been made at modifying conditions of media or incubation in efforts to improve culturable diversity.

The number of samples in provinces is imbalanced (for example, Tehran has 17 samples of trees in contrast to 4 in Alborz and 7 in Qom).

There can be an assessment of diversity within the tissues of all provinces and then an analysis of the importance of their distribution.

Reviewer #3: The study represents the diversity of endophytic fungi in Eucalyptus under various climatic conditions. The work is extensive and well presented both technically and scientifically.

Have you submitted the cultures in culture collection?

Have you submitted the ITS sequences in GenBank?

**********

6. PLOS authors have the option to publish the peer review history of their article (what does this mean?). If published, this will include your full peer review and any attached files.

If you choose “no”, your identity will remain anonymous but your review may still be made public.

**Do you want your identity to be public for this peer review?**  For information about this choice, including consent withdrawal, please see our Privacy Policy.

Reviewer #1: No

Reviewer #2: No

Reviewer #3: No

**********

[NOTE: If reviewer comments were submitted as an attachment file, they will be attached to this email and accessible via the submission site. Please log into your account, locate the manuscript record, and check for the action link "View Attachments". If this link does not appear, there are no attachment files.]

To ensure your figures meet our technical requirements, please review our figure guidelines: https://journals.plos.org/plosone/s/figures

You may also use PLOS’s free figure tool, NAAS, to help you prepare publication quality figures: https://journals.plos.org/plosone/s/figures#loc-tools-for-figure-preparation.

NAAS will assess whether your figures meet our technical requirements by comparing each figure against our figure specifications.

Attachment Submitted filename: Decsion for Revision - Mjaor Revision.docx

# Author comment: Author response to Decision Letter 1

**DOI:** 10.1371/journal.pone.0345700.r002

2 Feb 2026

Dear editor,

we are grateful for the very valuable reviewer comments. We have now thoroughly revised the manuscript addressing the comments.

With best regards,

Leila Ebrahimi, Ph.D.

Reviewers Comments:

Reviewer 1.

Major comments:

Introduction: The authors mention that fungal communities in Eucalyptus have been studied various parts of the world but not in Iran. This statement needs additional context and supporting references. I would suggest adding a brief summary of key findings from previous studies on fungal endophytes in Eucalyptus and clarify why examining these communities in Iran is important.

We have incorporated relevant findings from previous studies and expanded the introduction part on the importance of investigating Eucalyptus endophytes.

Discussion: The study focuses on fungal communities in leaves, fruits, stems, and branches; however, roots were not included. Root associated endophytic fungi play important roles in overall plant health and their communities may differ substantially from those in above-ground plant parts. Although it may not be feasible to add root samples at this stage, the authors should acknowledge this limitation and briefly discuss the ecological importance of root endophytes.

We have added both in discussion.

Lines 77 -78 and Lines 218-219: The control group used in this study is WA plates without plant samples. No fungal growth was observed on the control plates, indicating that the medium and handling conditions were sterile. This does not confirm the effectiveness of the surface-sterilization procedure, which would require plating the final rinse of water after surface sterilization or use tissue imprints.

Thank you for this observation. We have corrected it accordingly.

Minor comments:

Line 428, Line 500, Line 535: I have noticed sentences with informal tone in many sections of the manuscript. I would suggest revising the manuscript for a more formal and academic tone.

Corrected.

Line 528: “Genera like Cladosporium and Penicillium were found everywhere…”. Please be more specific about what ‘everywhere’ refers to (eg. all sampling sites, all plant tissues, or all climates).

Corrected.

Including a map of Iran showing the sampling locations as a supplementary figure would be helpful for the readers.

We have added a map showing the sampling locations as S1 Figure.

Reviewer 2:

Include in a brief table the climatic and geographic parameters of each province, such as mean annual precipitation -MAP, mean annual precipitation-MAT, humidity, and elevation, preferably from the same period of time as the sampling. Employ these variables to better underpin the arid–humid gradient and place the interpretation of "macro-climate" into a more quantitative context than simple descriptive terms.

We have added a supplementary table (S1 Table) with information for each province.

In the Discussion, make sure to differentiate those factors that could be reasonably attributed to climate from those that may also reflect stand history, host genetic diversity, air quality, or the deployment of agrochemicals; currently, climate is sometimes posed as just about the only driver.

Thank you for this important comment, which encourages greater precision in attributing ecological drivers. We have revised the Discussion to acknowledge that factors such as stand history, host genetics, and local conditions can, in theory, influence endophyte communities.

Improvise the figures with clarity of visibility.

Thanks. We improved the quality of the figures.

All isolates of WA → PDA have only culturable isolates, which automatically results in underrepresentation of most endophyte species. It should be noted whether any attempt has been made at modifying conditions of media or incubation in efforts to improve culturable diversity.

This is an excellent idea; however, we have not implemented it.

The number of samples in provinces is imbalanced (for example, Tehran has 17 samples of trees in contrast to 4 in Alborz and 7 in Qom). There can be an assessment of diversity within the tissues of all provinces and then an analysis of the importance of their distribution.

Thank you for raising this valid point regarding the uneven sampling across provinces. We acknowledge the imbalance, which resulted from the natural, patchy distribution of Eucalyptus plantations across Iran's varied climates. We chose to sample all available, healthy stands within our target climatic gradient, prioritizing ecological representativeness over numerical parity.

We wish to assure you that the diversity metrics central to our conclusions are robust to this sampling heterogeneity for the following reasons:

Use of sample-size corrected indices: our analysis relies on diversity indices explicitly designed to be comparable across different sampling efforts. The Shannon-Wiener (H') and Simpson's (1-D) indices incorporate species richness and relative abundance, while Pielou's Evenness (J') measures the distribution of individuals. Crucially, the Chao1 estimator was used to estimate total species richness, a non-parametric metric that is reliable for smaller samples as it is based on the frequency of rare species.

Focus on proportional colonization and relative patterns: our key findings are based on proportional metrics (Colonization Frequency, Relative Abundance) and consistent patterns (e.g., the universal leaf > branch > fruit hierarchy; the climate-driven richness gradient from humid to arid provinces). These patterns are evident in the standardized data and are not an artifact of varying tree counts.

Consistency with ecological expectation: the observed pattern—highest diversity in humid Mazandaran and lowest in arid Alborz/Qom—aligns strongly with established global principles of microbial biogeography. The fact that this clear gradient emerges despite sampling constraints reinforces, rather than weakens, the primary role of climate as a driver.

Reviewer 3:

The study represents the diversity of endophytic fungi in Eucalyptus under various climatic conditions. The work is extensive and well-presented both technically and scientifically.

Have you submitted the cultures in culture collection? Have you submitted the ITS sequences in GenBank?

Yes, we have deposited the cultures in a collection and the corresponding sequences in NCBI, with all related accession numbers and strain data provided in S2 Table.

Thank you so much again for your invaluable comments.

Best Regards.

Attachment Submitted filename: reviewers response.docx

# Peer review: Decision Letter 1

**DOI:** 10.1371/journal.pone.0345700.r003

**Authors:** Phalisteen Sultan, Phalisteen Sultan

10 Mar 2026

Manuscript No. : PONE-D-25-55331

Title : "Biodiversity of Eucalyptus endophytic fungi across different climates in Iran"

Dear Dr. Leila Ebrahimi,

We’re pleased to inform you that your manuscript has been judged scientifically suitable for publication and will be formally accepted for publication once it meets all outstanding technical requirements. Based on the recommendation & comments received after revision 1, your manuscript is Accepted for publication after you will carry out below given minor corrections as suggested by the reviewer.

Below, please find the reviewers' comments for your perusal. You are informed to also check the website for possible reviewer attachment(s). While submitting, please check the filled in author data carefully and update them if applicable - they need to be complete and correct in order for the revision to be processed further.

Within one week, you’ll receive an e-mail detailing the required amendments. When these have been addressed, you’ll receive a formal acceptance letter and your manuscript will be scheduled for publication.

An invoice will be generated when your article is formally accepted. Please note, if your institution has a publishing partnership with PLOS and your article meets the relevant criteria, all or part of your publication costs will be covered. Please make sure your user information is up-to-date by logging into Editorial Manager at Editorial Manager® and clicking the ‘Update My Information' link at the top of the page. For questions related to billing, please contact billing support.

If your institution or institutions have a press office, please notify them about your upcoming paper to help maximize its impact. If they’ll be preparing press materials, please inform our press team as soon as possible -- no later than 48 hours after receiving the formal acceptance. Your manuscript will remain under strict press embargo until 2 pm Eastern Time on the date of publication. For more information, please contact onepress@plos.org.

Kind regards,

Academic Editor

PLOS One

Additional Editor Comments (optional):

**Reviewer Comments:**

***Lines 68 and 70:***  Please check if the reference #25 is the right citation. Reference #25 (Fisher et al, 1943) discusses about relationship between species richness and number of individuals in animal population. I could not find any reference on *E. nitens, Botryosphaeria and Cytospora* . Please verify this and add the appropriate citation.

# Editor report: Acceptance letter

**DOI:** 10.1371/journal.pone.0345700.r004

**Authors:** Phalisteen Sultan, Phalisteen Sultan

PONE-D-25-55331R1

PLOS One

Dear Dr. Ebrahimi,

I'm pleased to inform you that your manuscript has been deemed suitable for publication in PLOS One. Congratulations! Your manuscript is now being handed over to our production team.

At this stage, our production department will prepare your paper for publication. This includes ensuring the following:

* All references, tables, and figures are properly cited

* All relevant supporting information is included in the manuscript submission,

* There are no issues that prevent the paper from being properly typeset

You will receive further instructions from the production team, including instructions on how to review your proof when it is ready. Please keep in mind that we are working through a large volume of accepted articles, so please give us a few days to review your paper and let you know the next and final steps.

Lastly, if your institution or institutions have a press office, please let them know about your upcoming paper now to help maximize its impact. If they'll be preparing press materials, please inform our press team within the next 48 hours. Your manuscript will remain under strict press embargo until 2 pm Eastern Time on the date of publication. For more information, please contact onepress@plos.org.

You will receive an invoice from PLOS for your publication fee after your manuscript has reached the completed accept phase. If you receive an email requesting payment before acceptance or for any other service, this may be a phishing scheme. Learn how to identify phishing emails and protect your accounts at https://explore.plos.org/phishing.

If we can help with anything else, please email us at customercare@plos.org.

Thank you for submitting your work to PLOS ONE and supporting open access.

Kind regards,

PLOS ONE Editorial Office Staff

on behalf of

Dr. Phalisteen Sultan

Academic Editor

PLOS One
